# Oxidative stress: a sex-specific cost of parental care

**DOI:** 10.1242/bio.062571

**Published:** 2026-06-24

**Authors:** Norma Martínez-Lendech, Ivette Marai Villa-Villaseñor, Luis Mendoza-Cuenca, Jorge Contreras-Garduño

**Affiliations:** ^1^ Laboratorio de Ecología Evolutiva, Escuela Nacional de Estudios Superiores Unidad Morelia, Universidad Nacional Autónoma de México, Morelia, Michoacán 58190, Mexico; ^2^ Licenciatura en Turismo, Instituto Tecnológico Superior del Oriente del Estado de Hidalgo, Tecnológico Nacional de México, Apan, Hidalgo 43900, Mexico; ^3^ Laboratorio de Ecología del Comportamiento, Facultad de Biología, Universidad Michoacana de San Nicolás de Hidalgo, Morelia, Michoacán 58030, Mexico

**Keywords:** Fish, Ecophysiology, Endangered species, Endemic species, Cichlids

## Abstract

Parental care involves post-zygotic behaviors that promote offspring survival but can diminish parental survival and future reproductive potential. Although care is typically shared, males and females often differ in the nature and intensity of care provided. In the wild, we characterized pair formation and parental care (nesting and fry care) in *Herichthys bartoni*. Behavioral investment was quantified as the proportion of observation time allocated to each activity. Fin tissue samples were collected to evaluate oxidative stress across reproductive stages. We observed stage- and sex-specific behavioral differences. During pair formation, both sexes fed and engaged in synchronized swimming, with no significant sex differences. During nesting, females allocated a significantly greater proportion of observation time to nest cleaning and egg aeration, whereas no sex differences were found during fry care. Males were larger and exhibited higher total antioxidant levels, independent of body size. Antioxidant levels peaked during pair formation and fry care, with catalase activity being higher in females during fry care. No sex differences were detected in superoxide dismutase, hydrogen peroxide, or nitric oxide. These findings suggest that biparental care in *H. bartoni* involves distinct behavioral roles and physiological costs, with females bearing greater oxidative demands linked to their increased direct investment in offspring care.

## Teaser

**Summary:** This research shows that *Herichthys bartoni* mothers invest more than fathers in caring for offspring and face greater physical challenges, revealing the vital role of maternal care in survival.

## INTRODUCTION

Parental care comprises post-zygotic behaviors performed by one or both parents to enhance offspring survival, development, and reproductive success (Trivers, 1972; Clutton-Brock, 1991). This widespread phenomenon spans diverse taxa, from invertebrates to vertebrates, and includes activities such as egg attendance, nest maintenance, provisioning, and offspring protection (Smiseth, 2017; Haimson and Mizrahi, 2023). Care may be biparental, with both parents contributing, or uniparental, predominantly provided by one sex (Webb et al., 1999; Pilakouta et al., 2018). While parental care improves parental fitness, it carries substantial physiological costs, including reduced body condition, impaired immunity, and increased oxidative stress (Smith and Wootton, 1995; Steinhart et al., 2005; Wilson et al., 2012). These costs and benefits often differ between sexes, shaping diverse care strategies. Symmetrical costs may favor biparental care, whereas asymmetries promote sex-biased investment and sexual dimorphism in parental roles (Trivers, 1972; McNamara and Wolf, 2015; Sowersby et al., 2017).

Energetic demands in parental investment can deplete reserves, reducing survival and future reproduction, especially under environmental stress (Clutton-Brock, 1991; Steinhart et al., 2005). Elevated activity during care increases cellular metabolism and production of reactive oxygen species (ROS), potentially causing oxidative damage, accelerated aging, and lowered survival if not countered by antioxidant defenses (Costantini, 2008; Alonso-Alvarez and Velando, 2012). Although this link between oxidative stress and parental care has been reported (Alonso-Alvarez et al., 2004; Garratt et al., 2011; Wilson et al., 2012), few studies have examined this relationship in the wild considering both sexes across multiple reproductive stages.

Sex-specific physiological costs are documented; for example, males of *Taeniopygia guttata* exhibit higher oxidative stress than females (Alonso-Alvarez et al., 2004). Parental investment correlates with offspring size and oxidative status, influenced also by age-related factors (Alonso-Alvarez et al., 2010). In fishes, including many cichlids, both biparental and uniparental care strategies occur (Gross and Sargent, 1985). Cichlid care commonly involves substrate incubation, territorial defense, and complex social behavior, with females mainly providing direct offspring care and males focusing on territory defense (Jordan et al., 2021). Given these physiological costs, females may experience elevated oxidative stress.

**Fig. 1.**
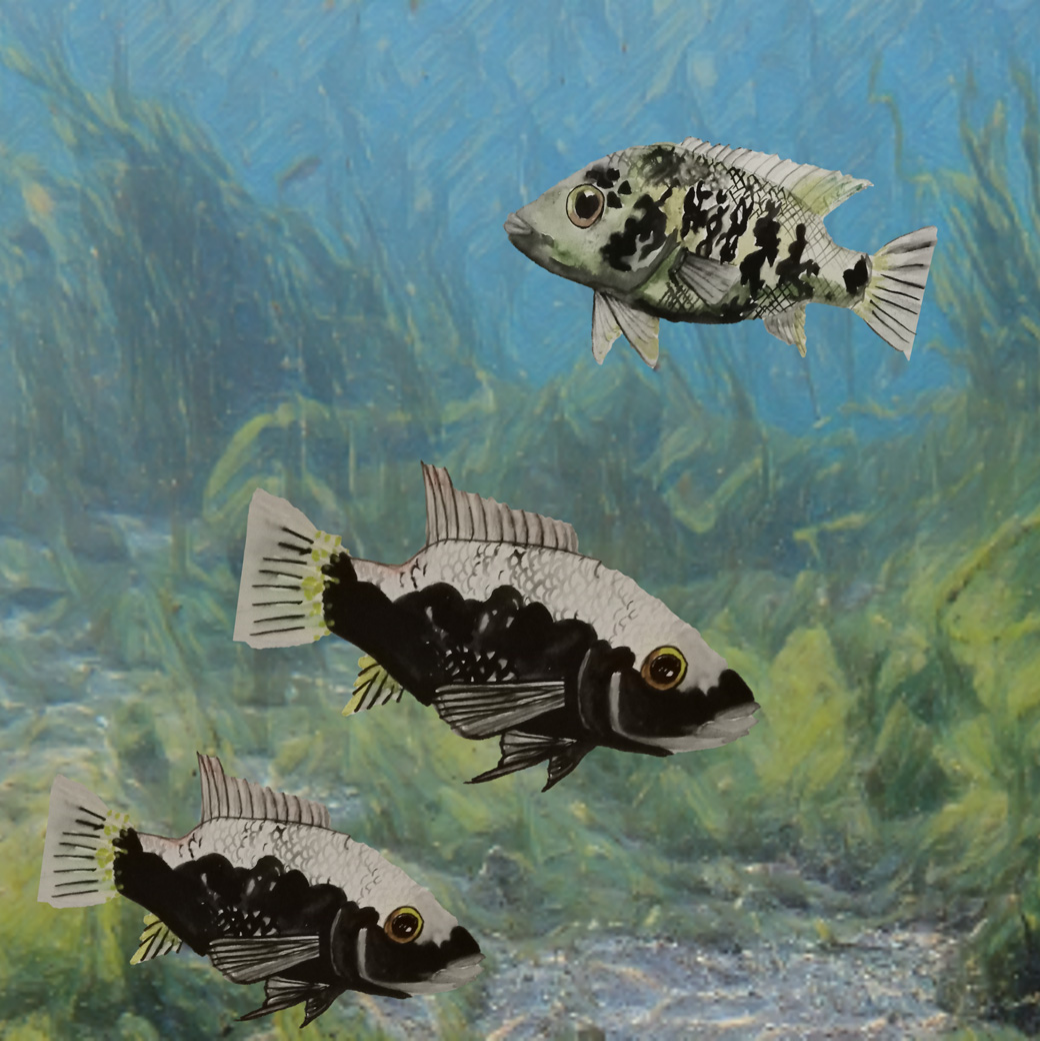
**Artwork showing a solitary male at the top, without nuptial coloration, and a pair below displaying the typical nuptial coloration, white (top of the body) and black.** Drawing by María de Lourdes Pérez Fosado.

Biparental care is considered ancestral in cichlids (Goodwin et al., 1998; Jordan et al., 2021). The cichlid *Herichthys bartoni*, endemic to Mexico's Verde River basin (De la Maza-Benignos and Lozano-Vilano, 2013), is classified as endangered [Secretaría de Medio Ambiente y Recursos Naturales (SEMARNAT), Norma Oficial Mexicana NOM-059-SEMARNAT-2010, https://www.dof.gob.mx; International Union for Conservation of Nature (IUCN), 2025, https://www.iucnredlist.org]. This primarily algivorous species undergoes distinct coloration changes linked to reproduction, with nuptial coloration developing in both sexes (Mejía et al., 2015; Fig. 1). We observed in the field that *H. bartoni*, together with *Herichthys labridens*, are the most abundant cichlid species in Media Luna Lagoon. The adult *H. labridens*, along with adults of two other introduced cichlid species from the genus *Oreochromis*, represent the only potential predators of juvenile *H. bartoni*. Hence, the offspring care seems to be important. Preliminary observations suggest sex-specific parental roles similar to those of other biparental cichlids (Snekser et al., 2011). The current study seeks to assess parental behavior and the physiological costs associated with parental care in *H. bartoni*, with particular emphasis on sex-specific differences in resistance to oxidative stress. Given that *H. bartoni* is endemic to the study area, understanding its behavioral and reproductive biology is vital for informing effective conservation strategies. We hypothesize that parental care contributes to increased oxidative stress and exhibits sexual dimorphism. We predict that *H. bartoni* will demonstrate sex-specific differences in parental investment, with males primarily responsible for defending the territory and nest, while females focus on direct care of the offspring. Furthermore, we predicted that oxidative stress would escalate with parental care, with the sex investing more in parental care incurring greater oxidative stress costs compared to the sex investing less.

## RESULTS

### Behavioral observations

**Fig. 2.**
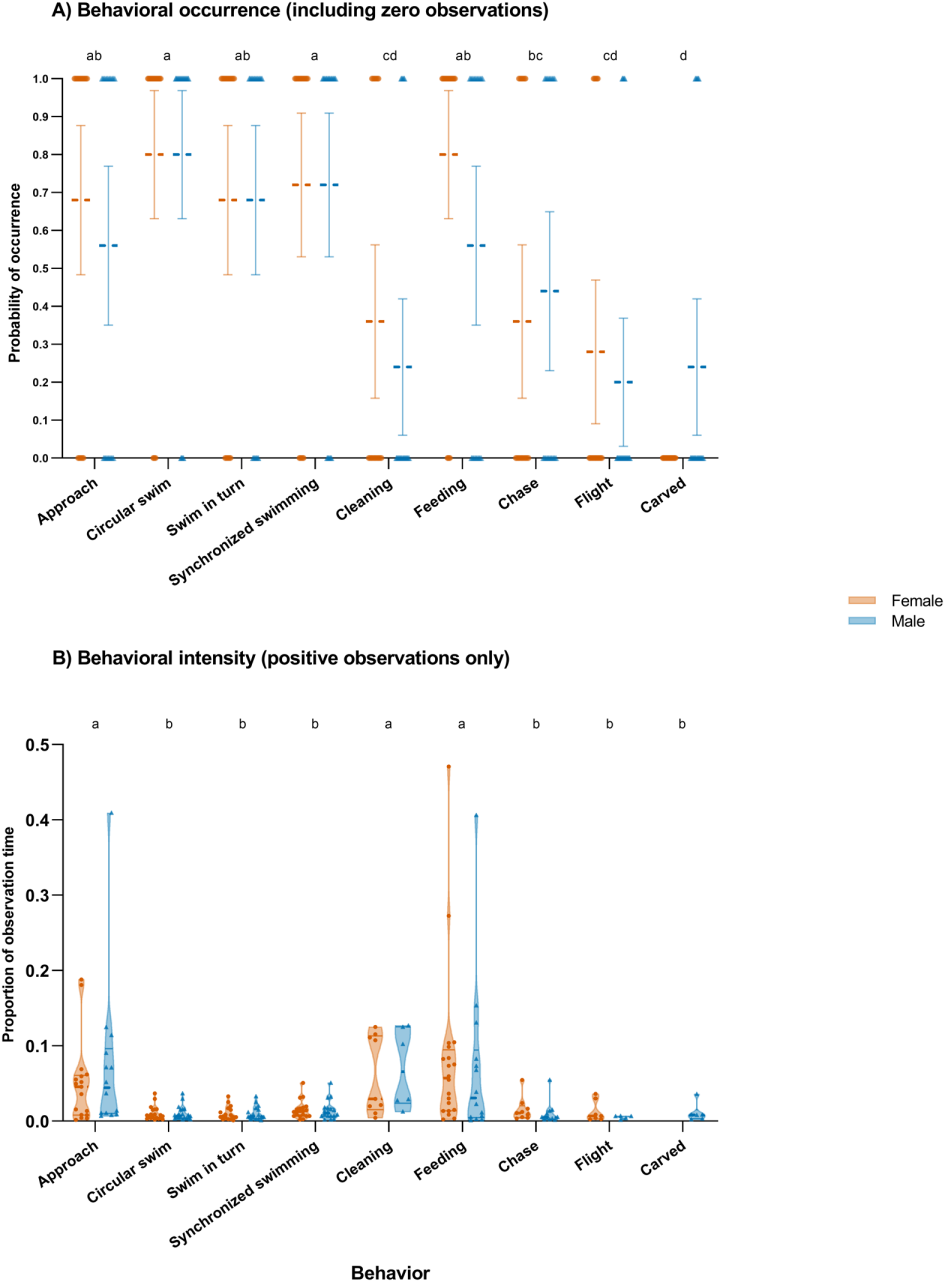
**Intersexual differences in behaviors performed by *H. bartoni* pairs during the pair formation stage.** (A) Probability of behavior occurrence, including zero observations. (B) Proportion of total observation time allocated to each behavior when it was expressed (positive observations only). Colors represent sex (female=orange, male=blue). Symbols denote individual observations; bars and whiskers indicate estimated marginal means±95% confidence intervals. Because no significant sex differences or sex×behavior interactions were detected, comparisons among behaviors were averaged across sexes. Different letters denote significant differences among behaviors (Tukey-adjusted comparisons, *P*<0.05).

Behavioral observations were cataloged in an ethogram comprising three stages: pair formation, nesting, and fry care (Table 1). Feeding, being chased by another individual (intersexual chase), synchronized swimming, circling, turning, and crowding were observed exclusively during pair formation in both females and males. Carving behavior was recorded only in males (Fig. 2A; Table S1).

**Table 1.** An ethogram presenting the differences in behaviors performed by the *H. bartoni* pair during the three stages of the reproductive event

Behavior	Reproductive stage	Description
	Pair formation	
Chase	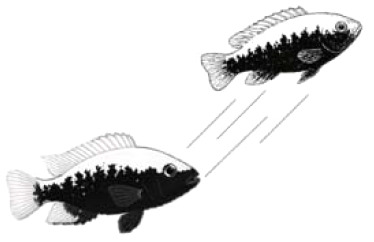	The male or female swims toward another individual to scare it away.
Confrontation	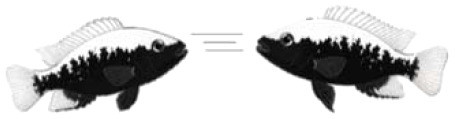	The male or female engages in direct head-on contact with another fish.
Flight	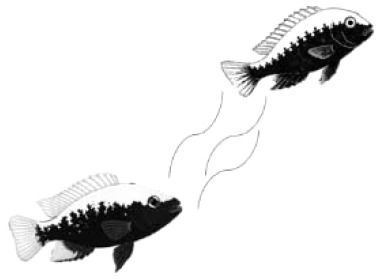	The male or female rapidly swims away from another individual.
Synchronized swimming	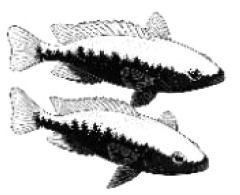	The male and female swim in parallel, exhibiting synchronized movement.
Circular swim	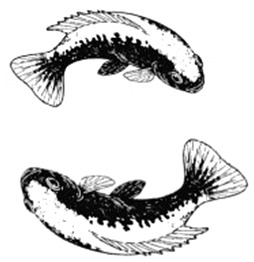	The male and female exhibit synchronized turning behavior, each following the caudal fin of the other.
Swim in turn	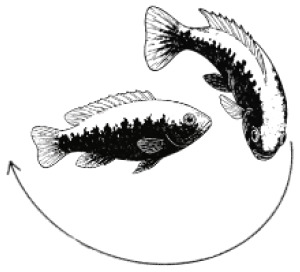	Male circles the female, whereas the female remains stationary in the water.
Approach	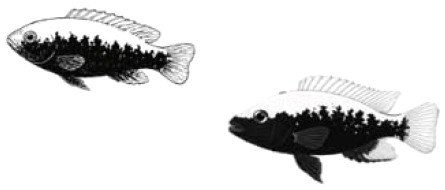	One individual swims toward and follows the mate (the male follows the female or the female follows the male).
Cleaning	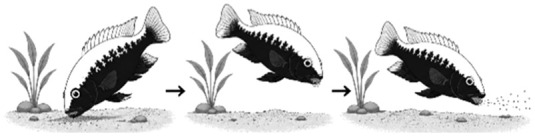	The individuals lift the substrate with their mouths, move away from the site, and expel the substrate. They appear to be cleaning the spawning site or its surroundings, perhaps to prepare the nest.
Feeding	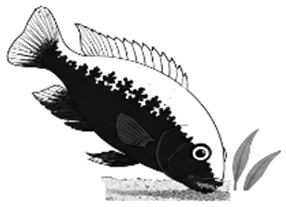	The individuals consume food directly from the substrate using their mouths.
Carved	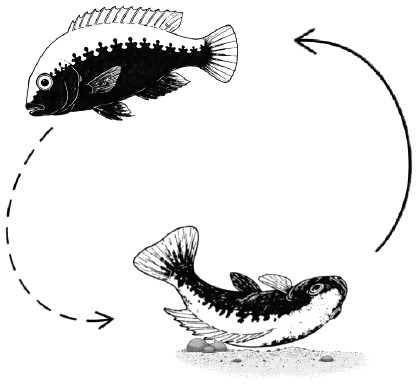	The male rubs its body against the substrate.
	Nesting period	
Spawn	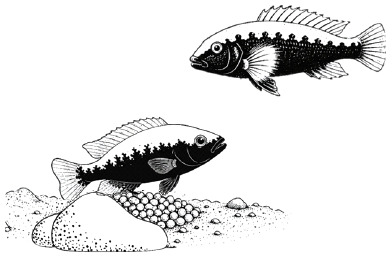	The individuals move slowly over the spawning site, likely depositing eggs and sperm.
Chase	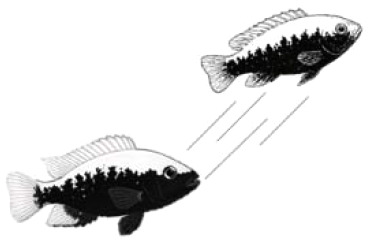	The male or female swims toward another individual to scare it away.
Inspection	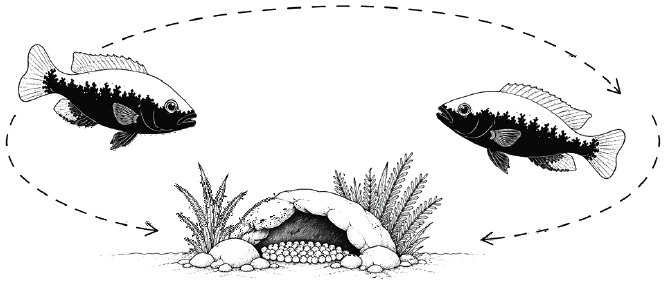	The individuals swim around the spawning site, likely monitoring for potential intruders nearby.
Aeration	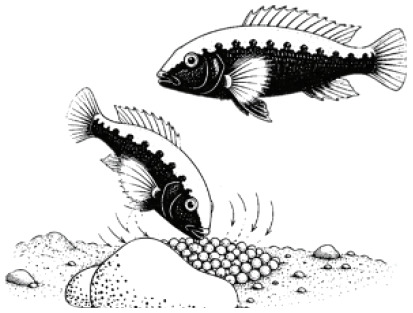	Individuals swim above the eggs and use their pectoral fins to fan water over them.
Cleaning	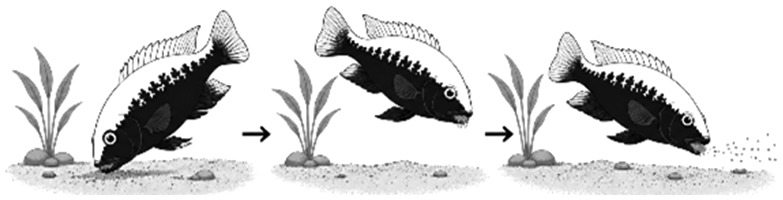	The individuals lift the substrate with their mouths, move away from the site, and expel the substrate. They appear to be cleaning the spawning site or its surroundings, perhaps to prepare the nest.
Changing the location of the eggs	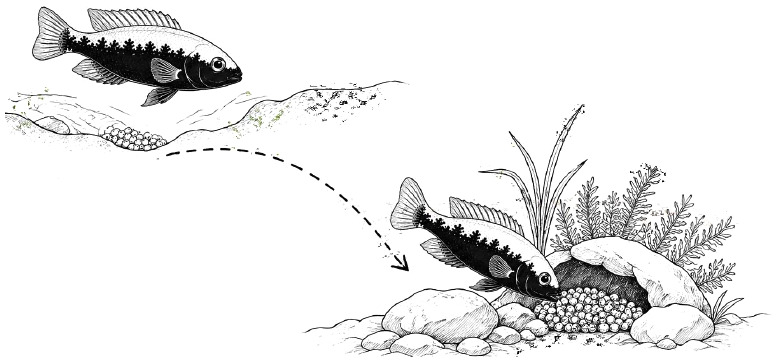	Females relocate the eggs from the initial spawning site to a secondary location that appears to offer greater protection.
	Fry care	
Chase	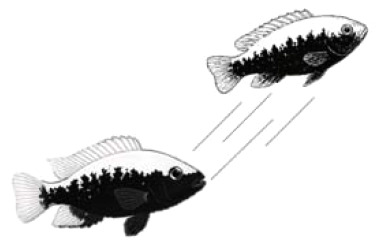	The male or female swims toward another individual to scare it away.
Confrontation	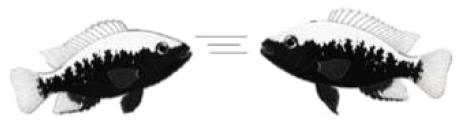	The male or female has head-on contact with another fish.
Inspection	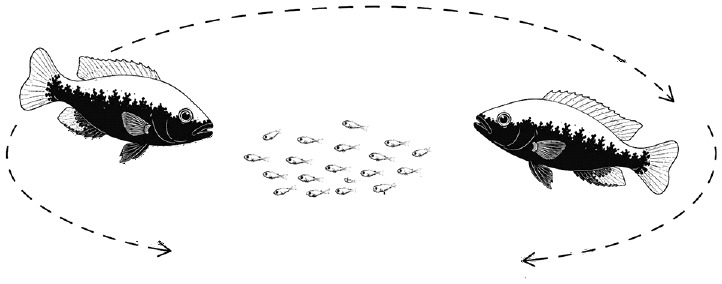	The individuals swim around the spawning site, likely monitoring for potential intruders nearby.
Leave	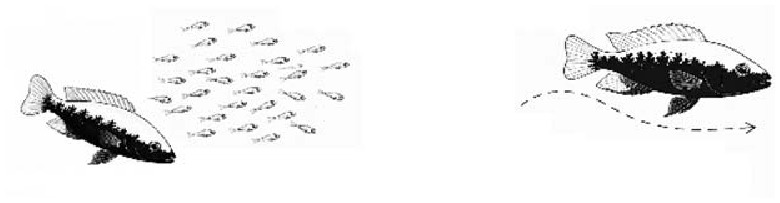	The male departs from the female and the responsibility of caring for the young but frequently returns.
Custody	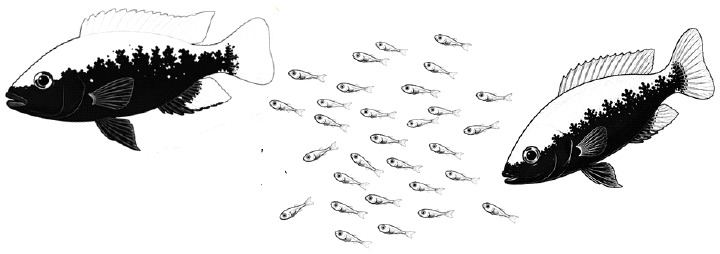	Both parents watch over the fry to prevent intruders from approaching.
Guard	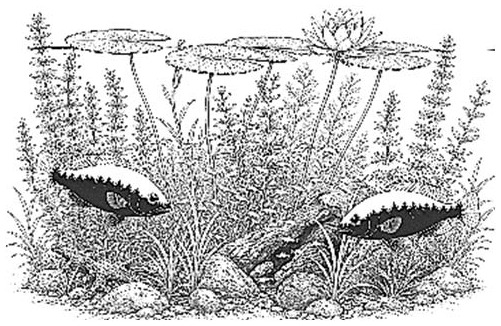	Both parents protect the fry among aquatic vegetation.

No significant differences between females and males were detected in the probability of occurrence or in the proportion of observation time allocated to these behaviors during pair formation (Fig. 2A; Table S2). Pair identity explained approximately 29.6% of the variance in behavior occurrence, whereas its contribution to the variance in behavioral intensity was practically negligible. However, several behaviors differed across stages. In particular, circling, synchronized swimming, and crowding were among the most frequent behaviors, whereas carving, flight, and cleaning occurred less often. Behavioral intensity followed a consistent pattern, with feeding, cleaning, and approach accounting for a greater proportion of observation time compared with the remaining behaviors (Fig. 2B; Table S2). For most of the observation period, individuals swam continuously at the same location (84% of females and 87% of males; Table S1). The complementary zero-inflated beta mixed model supported the same overall interpretation, with no evidence of consistent sex differences in behavioral investment during pair formation (Table S2).

**Fig. 3.**
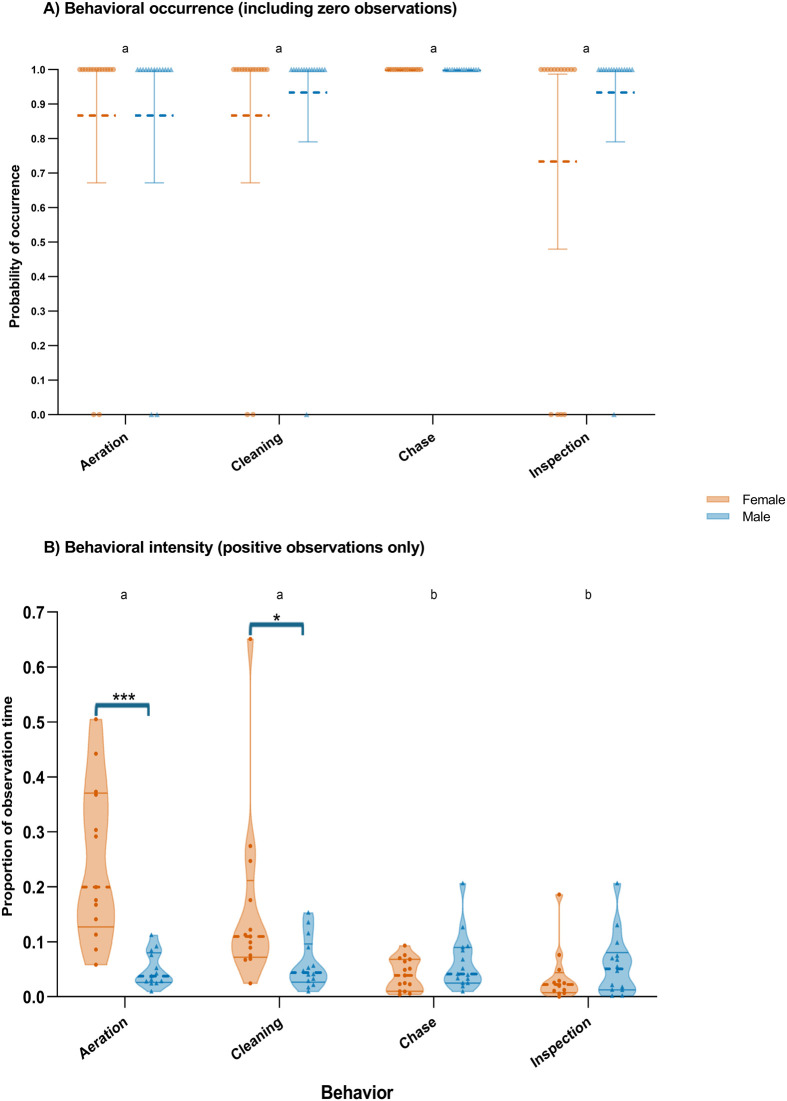
**Intersexual differences in behaviors performed by *H. bartoni* pairs during the nesting period.** (A) Probability of behavior occurrence, including zero observations. (B) Proportion of total observation time allocated to each behavior when it was expressed (positive observations only). Colors represent sex (female=orange, male=blue). Symbols denote individual observations; bars and whiskers indicate estimated marginal means±95% confidence intervals. Because no significant sex differences were detected in the probability of occurrence, comparisons among behaviors in A were averaged across sexes. In contrast, behavioral intensity differed between sexes for specific behaviors. Different letters denote significant differences among behaviors (Tukey-adjusted comparisons, *P*<0.05), and blue brackets indicate significant differences between females and males within each behavior (Šidák-adjusted comparisons, *P=0.011; ***P<0.0001).

During the nesting period, both females and males performed parental care behaviors associated with egg maintenance and nest defense. These activities primarily included aeration, cleaning, inspection of the spawning site, and chasing potential intruders (Table 1). No significant sex differences were detected in the probability of occurrence of these behaviors (Fig. 3A; Table S3). However, behavioral intensity differed between sexes for specific parental care behaviors. Females allocated a significantly greater proportion of observation time than males to aeration (*z*=5.04, *P*<0.001) and cleaning (*z*=2.51, *P*=0.01; Fig. 3B; Table S3), whereas no significant sex differences were observed for inspection or chase behaviors. Egg relocation was observed only in females, who also allocated a greater proportion of observation time to spawning than males (Table S1). Overall, cleaning and aeration accounted for a greater proportion of observation time than other activities, such as changing, spawning, inspection, and chasing (Fig. 3B; Table S1). Pair identity explained approximately 42.1% of the variance in behavior occurrence, whereas its contribution to the variance in behavioral intensity was negligible. The complementary zero-inflated beta mixed model produced qualitatively consistent results (Table S3).

**Fig. 4.**
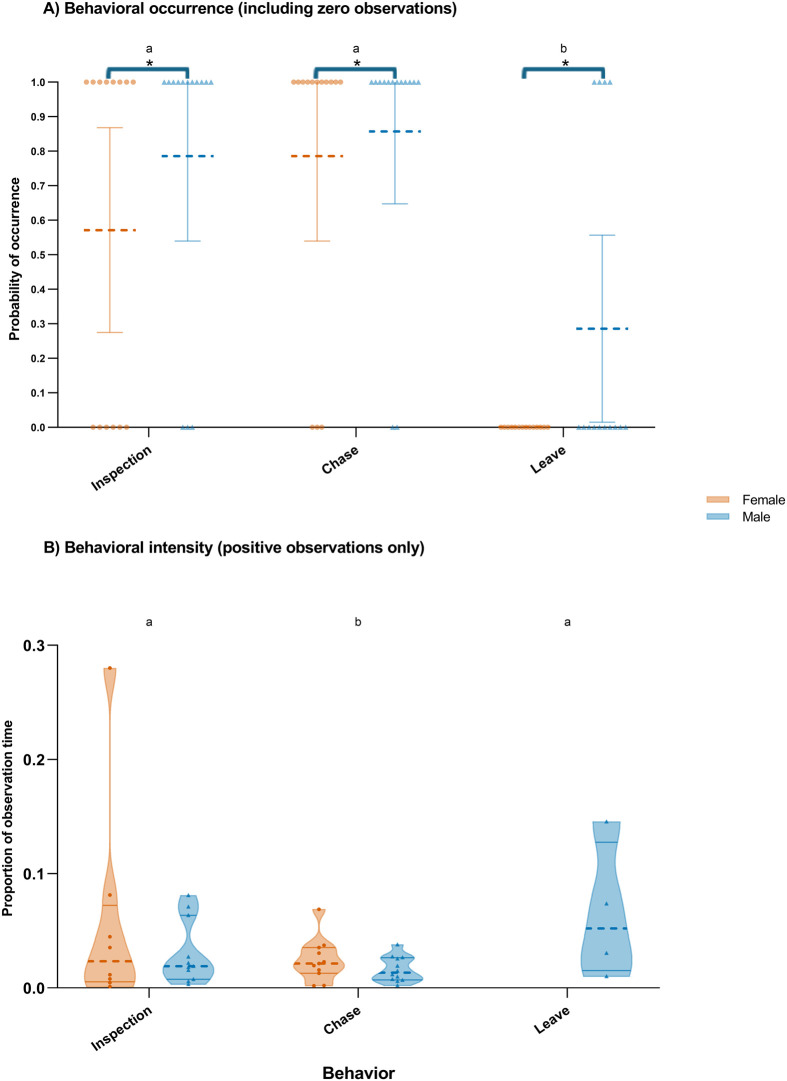
**Intersexual differences in behaviors performed by *H. bartoni* pairs during fry care.** (A) Probability of behavior occurrence, including zero observations. (B) Proportion of total observation time allocated to each behavior when it was expressed (positive observations only). Colors represent sex (female=orange, male=blue). Symbols denote individual observations; bars and whiskers indicate estimated marginal means±95% confidence intervals. Different letters denote significant differences among behaviors averaged across sexes (Tukey-adjusted comparisons, *P*<0.05), and blue brackets indicate significant differences between females and males within each behavior – inspection, chase and leave (Šidák-adjusted comparisons, *P=0.033).

During the fry care stage, pairs spent approximately 40% of the observation time in aquatic vegetation and 60% in unvegetated areas, consistently guarding their offspring. During this period, behaviors such as inspection and chasing were primarily directed toward fry protection and deterring potential intruders (Table 1). Males left parental care only during this stage and, in all cases, subsequently returned (Fig. 4A,B; Table S1). No significant sex differences were detected in behavioral intensity. However, sex significantly affected the probability of behavioral occurrence, with males exhibiting a higher probability of expressing behaviors than females (*z*=−2.12, *P*=0.03; Fig. 4A; Table S4). This effect was largely driven by the low-frequency behavior ‘leave’, which occurred disproportionately in males. Pair identity explained approximately 12.0% of the variance in behavior occurrence, whereas its contribution to the variance in behavioral intensity was negligible. The complementary zero-inflated beta mixed model produced qualitatively consistent results (Table S4).

**Fig. 5.**
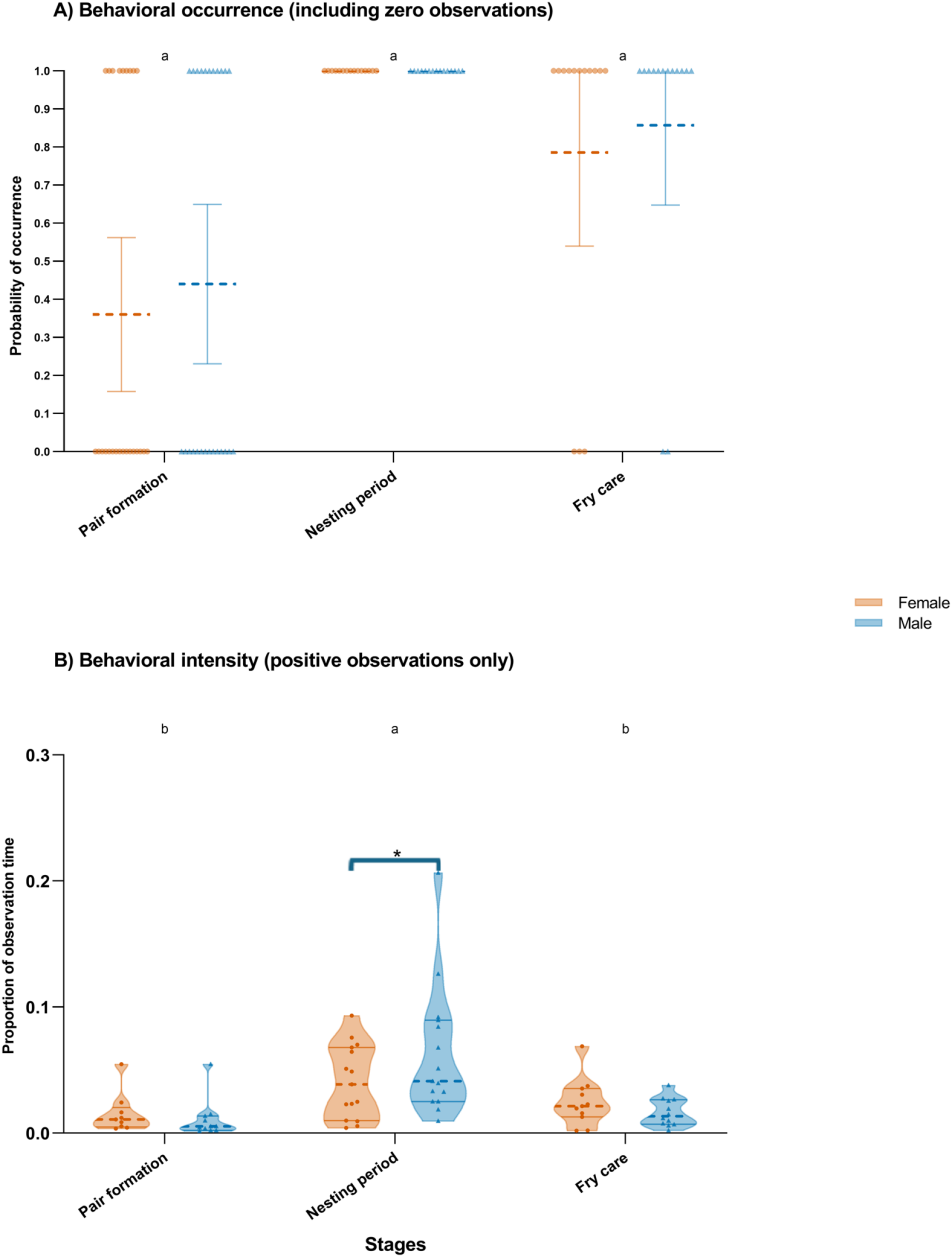
**Intersexual and across-stage differences in chasing behavior in *H. bartoni* during pair formation, nesting, and fry care.** (A) Probability of chase occurrence, including zero observations. (B) Proportion of total observation time allocated to chasing when it was expressed (positive observations only). Colors represent sex (female=orange, male=blue). Symbols denote individual observations; bars and whiskers indicate estimated marginal means±95% confidence intervals. Different letters denote significant differences among stages averaged across sexes (Tukey-adjusted comparisons, *P*<0.05), and blue bracket indicates significant differences between females and males within each behavior (Šidák-adjusted comparisons, *P=0.018).

Chasing was the only behavior exhibited by both females and males across all three stages. The probability of chase occurrence did not differ significantly among stages (Fig. 5A; Table S5). In contrast, the proportion of observation time allocated to chasing differed among stages, being significantly higher during the nesting period than during pair formation or fry care (Fig. 5B; Table S5). Significant sex differences in chase intensity were detected during the nesting period, with males allocating a greater proportion of observation time to chasing than females; however, no significant sex differences were observed during pair formation or fry care. This pattern was consistent with the complementary zero-inflated beta analysis. Pair-stage identity explained approximately 8.1% of the variance in the occurrence of chase behavior and 9.0% of the variance in its intensity.

**Fig. 6.**
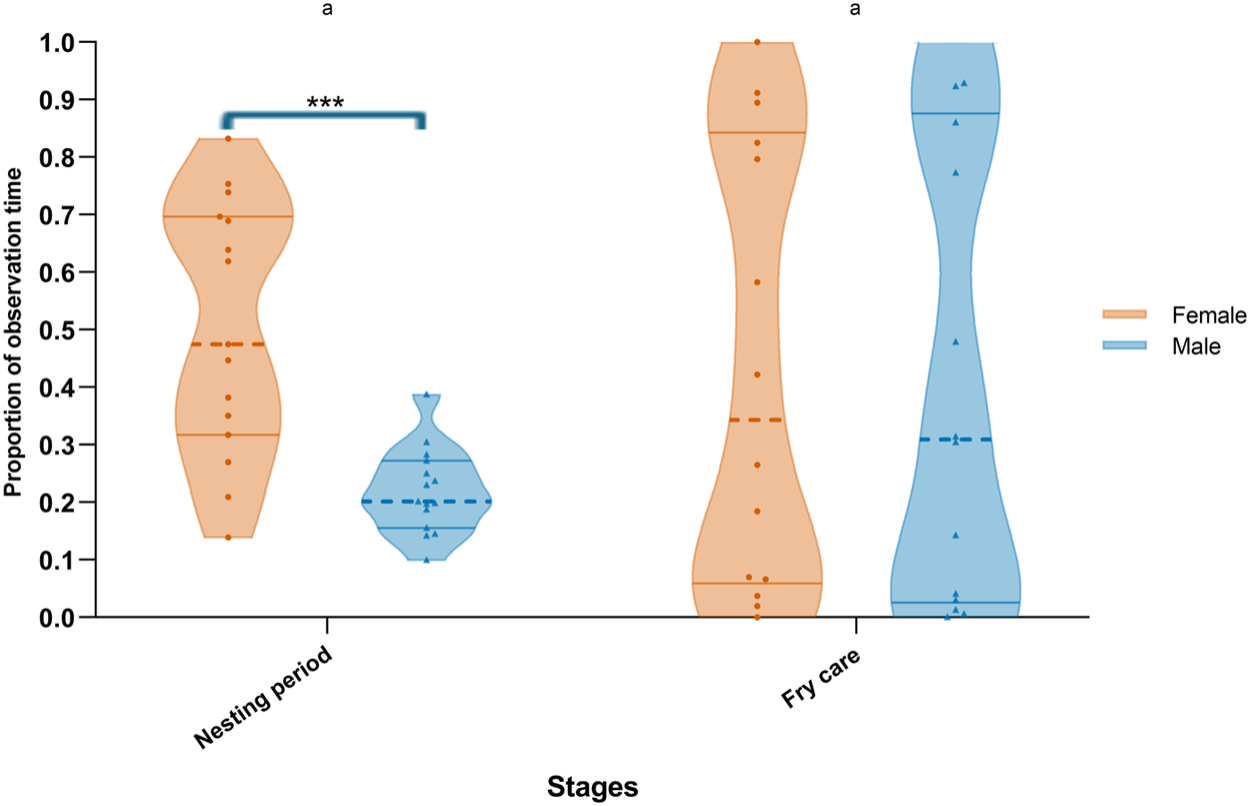
**Total proportion of observation time allocated to parental care behaviors by female and male *H. bartoni* during the nesting period and fry care.** Values were obtained by summing the proportions of observation time assigned to parental care behaviors within each stage. Colors represent sex (female=orange, male=blue). Symbols denote individual observations; bars and whiskers indicate estimated marginal means±95% confidence intervals. The same letter indicates no significant differences between stages (Tukey-adjusted comparisons, *P*>0.05), and blue bracket indicates significant differences between females and males within each stage (Šidák-adjusted comparisons, ***P<0.001).

Total parental care allocation differed between sexes as a function of reproductive stage (*z*=4.34, *P*<0.001; Fig. 6; Table S6). Pair-stage identity explained approximately 42.7% of the variance in total parental care allocation. During the nesting period, females allocated a greater proportion of their total observation time to parental care behaviors than males. In contrast, this sex difference was reduced during fry care. The total proportion of observation time devoted to parental care did not differ significantly between the nesting period and fry care in females, whereas males exhibited a relative increase in parental care during fry care compared with the nesting period.

### Sexual differences in oxidative stress

**Fig. 7.**
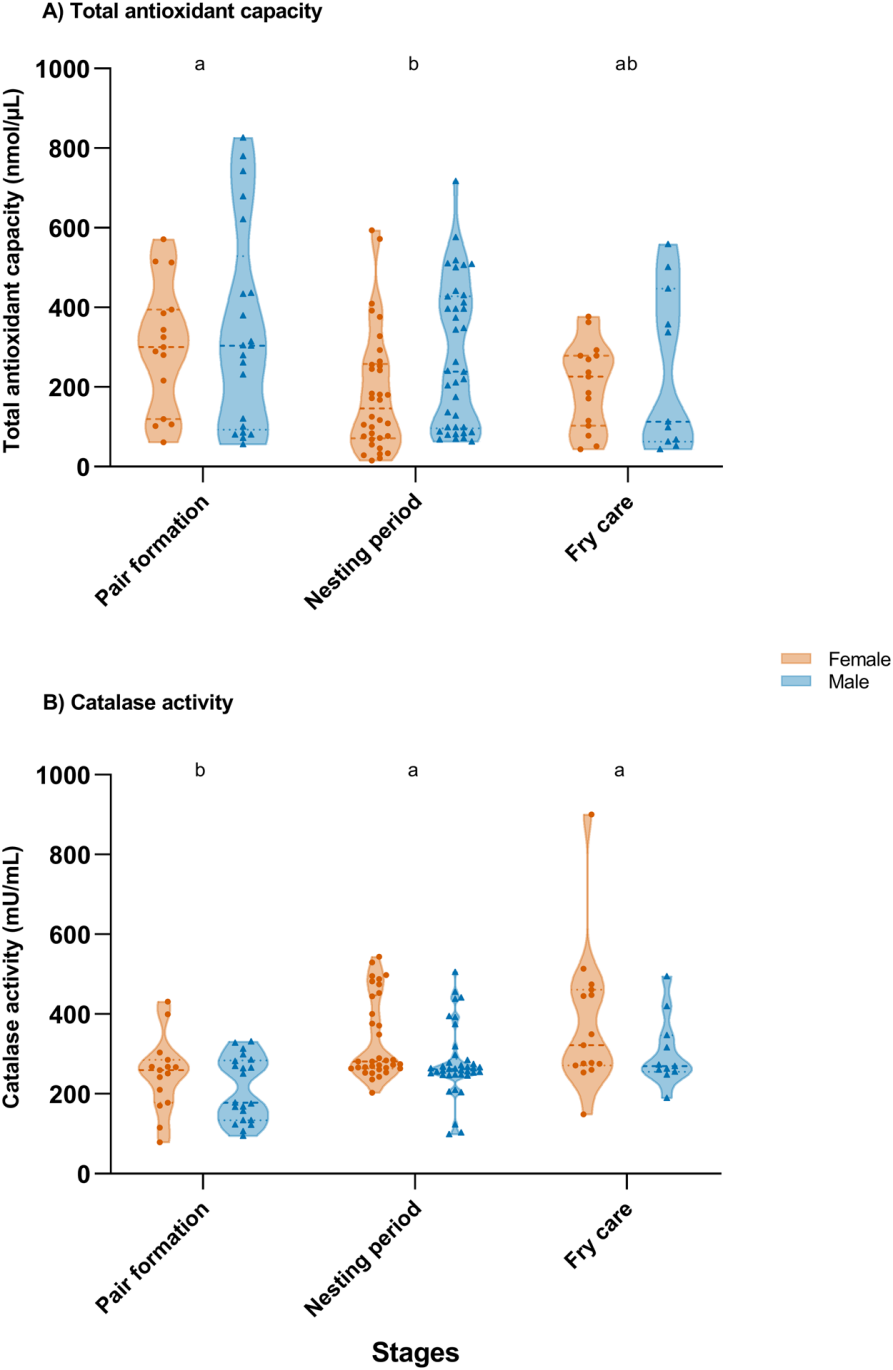
**Total antioxidants and CAT activity in female and male *H. bartoni* across reproductive stages.** (A) Total antioxidants. (B) CAT activity. Colors represent sex (female=orange, male=blue). Symbols denote individual observations; bars and whiskers indicate estimated marginal means±95% confidence intervals. Different letters indicate significant differences among stages based on Tukey-adjusted post hoc comparisons (*P*<0.05).

Significant sex differences in body size were found (*z*=−8.15, *P*<0.001): males were larger (9.09±0.17) than females (7.37±0.13). However, male and female sizes did not differ among parental care stages, and no sex×stage interaction was detected (Table S7). For total antioxidant levels (nmol/µl), significant sex differences were also observed (*z*=−2.124, *P*=0.033), with males exhibiting higher values (291.92±24.97) than females (216.78±18.76; Fig. 7A). Antioxidant levels further differed among stages (Fig. 7A; Table S7), with pair formation showing higher values (325.36±36.29) than both the nesting period (234.48±20.47) and fry care (219.73±29.83).

Catalase (CAT) activity (mU/ml) differed significantly between sexes (*z*=3.37, *P*<0.001): females exhibited higher activity (325.75±16.11) than males (261.73±10.87; Fig. 7B). Significant differences were also detected among reproductive stages (Table S7), with CAT activity being higher during fry care (346.70±29.26) than during both the nesting period (305.32±11.65) and pair formation (227.00±14.40).

No significant effects of sex, reproductive stage, or their interaction were detected for superoxide dismutase (SOD; % inhibition), hydrogen peroxide (H_2_O_2_; µM), or nitric oxide (NO; µM) (Table S7).

### Correlations between body size and oxidative stress in females and males

Spearman's rank correlation analyses revealed a significant positive relationship between female body size and total antioxidant content (*r*=0.41, *P*<0.001). In contrast, no significant relationship was found between male body size and total antioxidant content (*r*=0.21, *P*=0.08). Additionally, no significant relationships were detected between body size and CAT activity in either females (*r*=−0.23, *P*=0.06) or males (*r*=−0.018, *P*=0.88).

## DISCUSSION

Investment in parental care and its associated costs in *H. bartoni* were sexually dimorphic and differed across reproductive stages. Females allocated a greater proportion of parental care, and this greater effort was linked to the regulation of oxidative balance. In endemic and endangered species, such asymmetries in effort allocation may have population-level consequences, as any further increase in energetic demands (for example, due to habitat degradation or reduced resource availability) could negatively affect individual condition, reproductive success, and ultimately population dynamics and viability (Metcalfe and Alonso-Alvarez, 2010). In light of these findings, the observed sex differences in parental investment and the physiological costs of parental care are examined in detail below.

### Sex-specific differences in investment in parental behavior

According to our hypothesis, *H. bartoni* exhibits sex-specific differences in parental behavior that vary across reproductive stages. As predicted, such differences were observed and changed over the course of the reproductive cycle. At the beginning of each reproductive event (pair formation stage), males and females allocated similar proportions of observation time to foraging and mutual courtship behaviors. This is consistent with patterns described for other biparental cichlids, such as *Herichthys minckleyi*, *Amatitlania nigrofasciata*, and *Cichlasoma dimerus*, in which this stage is primarily devoted to consolidating the pair bond, establishing a territory, and coordinating reproduction rather than providing parental care (Itzkowitz et al., 2001; Oldfield et al., 2015; Birba et al., 2015; van Breukelen and Santangelo, 2021).

Once females laid their egg clutches, sex differences became evident throughout the nesting period, with females allocating a significantly greater proportion of observation time to nest cleaning and egg aeration. This pattern is consistent with observations in other biparental fishes, particularly substrate-brooding species, where females typically invest more in direct care, whereas males primarily contribute to territorial and nest defense (Keenleyside, 1991; Goodwin et al., 1998; Wisenden et al., 2008). For example, in *A. nigrofasciata*, *H. minckleyi*, and *Neolamprologus savoryi*, females invest more in direct offspring care, while males prioritize territorial defense and interactions with specific individuals (Balshine-Earn et al., 1998; Itzkowitz et al., 2001; Heg et al., 2005; Snekser et al., 2011). Substrate cleaning and continuous egg aeration are critical behaviors for reducing fungal infections and ensuring adequate embryonic oxygenation (Balshine, 2012), but they are energetically demanding, indicating greater parental effort by females during this stage. Thus, parental care is typically organized into sex-specific roles, even though both parents may perform all parental behaviors at different stages of offspring development (Snekser et al., 2011; Snekser and Itzkowitz, 2020). Differences in behavioral investment between males and females likely reflect sex-specific selection pressures on reproductive fitness (Leese and Blatt, 2021), as well as anatomical, hormonal, or physiological mechanisms that may facilitate or constrain the performance of particular parental behaviors (Snekser et al., 2011).

During the fry care stage, no significant sex differences were observed in the proportion of observation time allocated to parental behaviors. This result is similar to that reported in other biparental fishes, in which, once the young become mobile, both parents actively participate in their defense, care, and guidance (Wisenden et al., 2008). However, the similarity in behavioral investment at this stage does not necessarily imply equivalence in physiological costs. These costs may still differ due to variation in individual size (with males being larger) and because cumulative physiological costs can depend on the type, intensity, and duration of previous investments, including earlier nesting attempts and environmental variability (Clutton-Brock, 1991; Balshine et al., 2001). For instance, in *Amatitlania siquia*, although both sexes can assume each other's roles and pairs show highly coordinated care, males spend less time with offspring across the reproductive cycle, contributing less during the egg stage and more during the hatchling stage, whereas females maintain relatively constant levels of care (Snekser et al., 2011). An additional source of variation was pair identity. Pair identity accounted for a moderate proportion of the variance in behavioral occurrence but contributed negligibly to variation in behavioral intensity. These findings suggest that some pairs were more likely than others to exhibit parental behaviors; however, once these behaviors were expressed, the time allocated to them was primarily associated with behavioral category and reproductive stage rather than pair identity. This among-pair variation may reflect differences in pair coordination, reproductive experience, compatibility, or individual condition (Lehtonen, 2017; Snekser and Itzkowitz, 2020).

The sex- and stage-specific differences in parental behavior observed in *H. bartoni* suggest that biparental care entails differential physiological costs for each sex. In this context, oxidative stress represents a key physiological mechanism that may mediate trade-offs between investment in parental care and the maintenance of physiological conditions (Metcalfe and Alonso-Alvarez, 2010; Costantini, 2014). In the following section, we examine oxidative stress biomarkers to assess whether sex-specific differences in parental investment are mirrored by differences in oxidative status.

### The cost of reproduction associated with parental care involves oxidative stress

Parental care is associated with elevated metabolic demand, which can increase the production of ROS and, consequently, oxidative stress (Metcalfe and Alonso-Alvarez, 2010; Selman et al., 2012; Guindre-Parker et al., 2013; Speakman and Garratt, 2014; Cram et al., 2015a,b; Costantini, 2018). The level of oxidative stress in organisms depends on the type, intensity, and duration of their activities (Monaghan et al., 2009; Costantini, 2014). Low-intensity activities may increase ROS production without causing oxidative damage if antioxidant mechanisms are sufficient to counteract them. In contrast, more intense or prolonged activities can overwhelm these compensatory mechanisms (Speakman et al., 2015; Williams, 2018). In this study, oxidative stress biomarkers revealed physiological differences between sexes and among reproductive stages, suggesting that parental care in *H. bartoni* entails physiological costs and that, as in species such as the zebra finch (*T. guttata*), oxidative stress may increase with parental investment and brood size (Alonso-Alvarez et al., 2004). However, it remains to be tested whether differences in oxidative stress are also associated with other factors, such as age, number of nesting events, parental condition, and habitat characteristics.

The most pronounced changes in behavioral patterns in *H. bartoni* were observed during the nesting and fry care stages. During these stages, CAT activity showed a marked imbalance, with higher values in females, accompanied by reduced nonenzymatic antioxidant levels. CAT is a key enzyme in H_2_O_2_ detoxification, and increases in its activity are generally interpreted as a compensatory response to heightened oxidative stress (Costantini, 2019). This suggests that, although H_2_O_2_ levels did not differ among stages, females may be experiencing a greater oxidative load (Alonso-Alvarez et al., 2017). Similar results have been reported in other fishes, where expected increases in ROS are not always reflected in detectable accumulation of these compounds (Alonso-Alvarez et al., 2004; Costantini, 2014). One possible explanation is the efficient action of enzymatic antioxidant systems, particularly SOD, which converts superoxide radicals into H_2_O_2_ and thereby prevents their excessive accumulation (Birben et al., 2012; Halliwell and Gutteridge, 2015). Thus, coordinated activity among antioxidant enzymes may help maintain relatively stable levels of H_2_O_2_ and NO, even under high metabolic demand (Costantini and Verhulst, 2009).

Nonenzymatic antioxidants are often considered a first line of defense because they are less costly to maintain and depend largely on dietary intake and preexisting reserves (Costantini, 2014; Halliwell and Gutteridge, 2015; Pamplona and Costantini, 2011). During reproduction, however, these compounds may become limited due to reduced or suspended feeding and their transfer to offspring, particularly in females (Blount et al., 2004; Metcalfe and Alonso-Alvarez, 2010; Fletcher et al., 2013). This is consistent with our findings and may reflect both the physiological costs of egg production and the allocation of antioxidants to embryos as a protective mechanism against oxidative damage during early development (Surai et al., 2016). In this context, the increased CAT activity observed in *H. bartoni* likely reflects a compensatory upregulation of endogenous antioxidant defenses. Although the synthesis and regulation of CAT incur greater metabolic costs than the maintenance of nonenzymatic antioxidants, its high efficiency and specificity in H_2_O_2_ detoxification make it particularly relevant during periods of elevated metabolic demand, such as those associated with intense and prolonged parental care (Halliwell and Gutteridge, 2015; Costantini, 2019). This is especially important given that *H. bartoni* can reproduce multiple times per year, undergoing several periods of parental care.

Sexual size dimorphism is another factor that may contribute to the physiological differences observed between sexes. In *H. bartoni*, males reach larger body sizes than females, which is likely associated with enhanced performance in territorial defense, intraspecific competition, and reproductive success (Balshine, 2012). In many fish species, body size is positively correlated with competitive ability and access to resources, which can influence behavioral investment, body condition, and the physiological costs of reproduction (Anaya-Rojas et al., 2021). However, our analyses did not reveal a significant relationship between body size and CAT activity in either sex. This suggests that the increase in CAT activity observed in females cannot be explained solely by allometric differences but instead primarily reflects the intensity of reproductive investment and the type of parental care provided (Metcalfe and Alonso-Alvarez, 2010; Costantini, 2019). In contrast, a significant relationship between body size and total antioxidant levels was detected only in females, indicating that, in this sex, nonenzymatic antioxidant reserves are more closely linked to body size. Similar associations among body size, physiological condition, and antioxidant capacity have been reported in other vertebrates (Costantini and Verhulst, 2009; Selman et al., 2012; Beaulieu et al., 2014). The absence of such a relationship in males supports the notion that selective pressures and physiological trade-offs related to body size differ between sexes. Further studies will be required to determine whether the positive correlation observed in females is associated with higher fecundity, the production of larger gametes, or greater yolk allocation in larger females.

It is important to emphasize that the absence of differences in certain biomarkers does not necessarily indicate the absence of oxidative stress or oxidative damage. The assessment of oxidative status depends strongly on the type of biomarker and the tissue analyzed (Costantini, 2014; Speakman and Garratt, 2014). ROS not measured in this study may contribute to the increase in CAT activity, and oxidative responses can vary considerably among tissues. Consequently, the patterns detected in fin tissue may differ from those in more metabolically active organs such as the liver, muscle, or gonads (Selman et al., 2012; Costantini, 2019). For these reasons, it has been proposed that evaluations of oxidative stress should integrate multiple components of the redox system to allow more robust interpretation (Costantini and Verhulst, 2009; Alonso-Alvarez et al., 2010). Most previous studies of parental care in cichlids have focused primarily on behavior, with relatively few incorporating physiological biomarkers to quantify the costs of care (Goodwin et al., 1998; Snekser et al., 2011). In this context, the integration of oxidative stress biomarkers in the present study represents an important contribution, enabling a more comprehensive assessment of parental investment. We recognize the value of future studies that include multiple tissues or organs and analyze correlations according to age. Such efforts should be carefully designed with limited sample sizes, given the endangered status of *H. bartoni*.

### Conclusions

The results of this study indicate that parental care in *H. bartoni* entails sex-specific oxidative stress. Females allocated a greater proportion of observation time to direct offspring care, exhibited greater activation of enzymatic antioxidant defenses, and showed reduced levels of nonenzymatic antioxidants. These findings provide evidence of physiological trade-offs linked to reproductive investment. The absence of differences in ROS levels suggests that these costs do not necessarily manifest as cumulative oxidative damage, but rather as a redistribution of resources within the antioxidant system.

Taken together, these results highlight the importance of integrating behavioral and physiological perspectives to understand reproductive trade-offs and their implications for the conservation of endemic species such as *H. bartoni*. Populations of such species may be particularly vulnerable to habitat degradation, reduced food availability, or changes in water quality that elevate physiological costs. Even if the increase in oxidative stress observed across the care period in *H. bartoni* does not solely reflect intersexual differences in parental effort, it may nonetheless reduce parental survival or future reproductive performance, ultimately affecting population persistence. Understanding the physiological limits of parental care is therefore essential for predicting how endemic species will respond to environmental change and for designing conservation strategies that support both reproductive success and long-term population viability.

## MATERIALS AND METHODS

### Study area

**Fig. 8.**
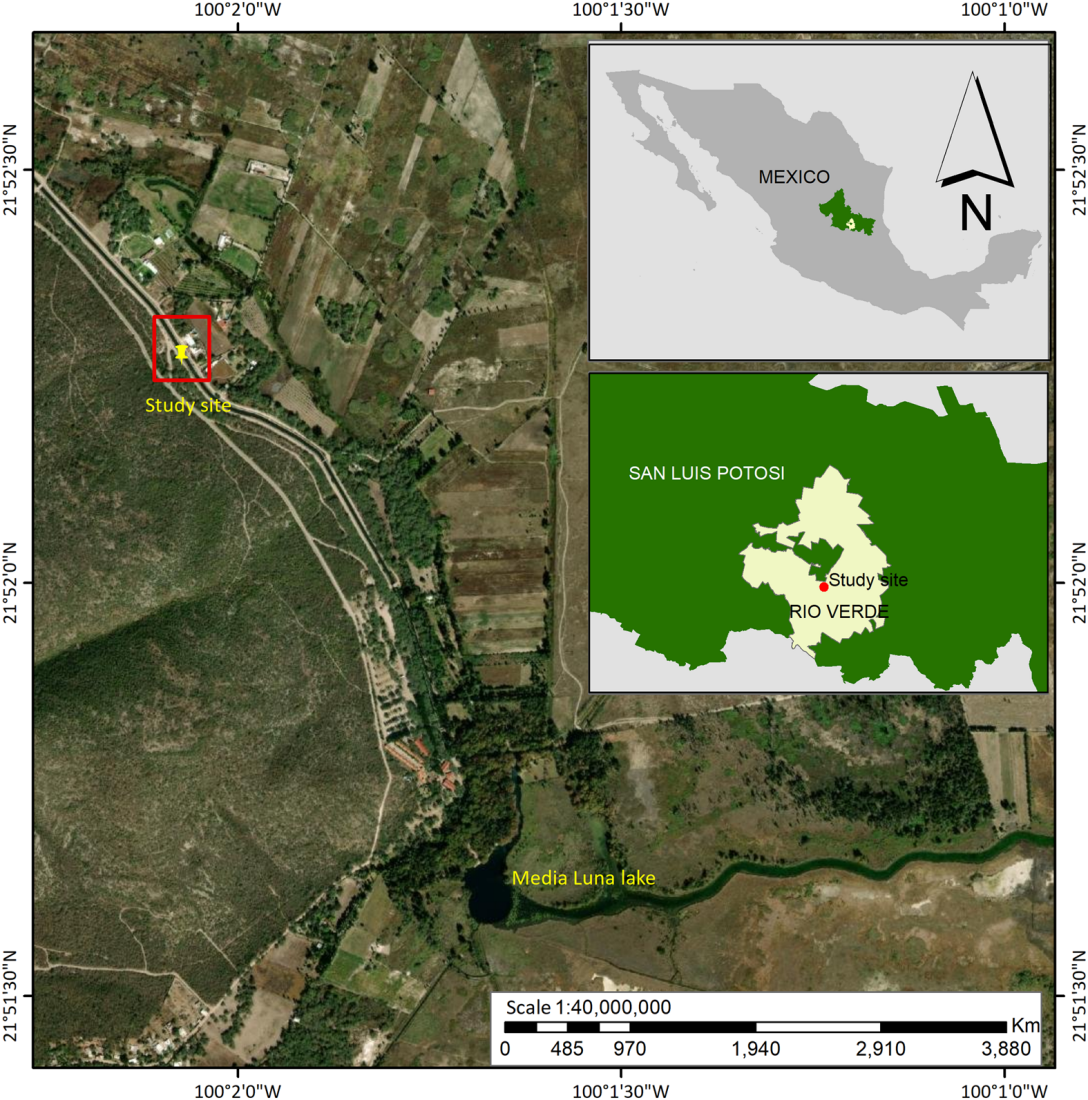
Site where the behavioral recordings and *H. bartoni* species sample collection were made.

Media Luna Lagoon is a spring situated in the municipality of Rioverde, San Luis Potosí, Mexico. This site is designated as a Protected Natural Area and is located between the following coordinates: 21° 51′ 34.29″–21° 51′ 47.67″ N and 100° 00′ 47.67″–100° 01′ 42.72″ W (Palacio-Nuñez et al., 2009; Fig. 8).

### Ethical statements

Behavioral observations and sample collection of *H. bartoni* were reviewed and authorized by an Animal Rights Committee under license number SEMARNAT: SGPA/DGVS/03925/22.

### Behavior observations

We recorded behavioral observations of emerging and established pairs of *H. bartoni* (including feeding, courtship, territorial defense, and parental care) throughout the reproductive period, which was divided into three stages: pair formation, nesting, and fry care.

During the pair formation stage, field videos were recorded more frequently but for shorter durations, as the fish exhibited rapid movement and were often lost from view. In the nesting period, pairs remained close to the egg site; preliminary observations indicated that 15-min video sessions were adequate to capture parental care behaviors. Similarly, during the fry care stage, the duration of observations was reduced, as both parents and fry sought refuge in the brush for protection. Video recordings were conducted at Media Luna Lagoon. During the pair formation stage, a total of 25 videos were collected (*n*=25 pairs), with durations ranging from 22 to 869 s. For the nesting period, 15 videos were recorded (*n*=15 pairs), lasting between 491 and 1686 s. In the fry care stage, 14 videos were collected (*n*=14 pairs), with durations varying from 79 to 975 s. The variation in recording times was attributed to individuals swimming continuously and losing visual contact, either due to increased distance or to them seeking refuge among lilies. Following the behavioral observations of each pair, we captured the fish to take measurements and clip a section of the fin from each individual for oxidative stress analysis (details provided below). This procedure enabled us to identify fish previously observed, thereby preventing their reuse and avoiding pseudo-replication in our sampling. In total, we sampled 54 unique pairs.

Given the limited and largely anecdotal information available for this species, an ethogram was constructed based on established descriptions of cichlid behavior (Baerends and Baerends, 1950) and field observations. Focal recordings were analyzed by pausing the video at 1-s intervals to quantify the duration of each activity. The proportion of time allocated to each behavior by females, males, or both parents was then compared, and behavioral patterns were systematically characterized. Behaviors were subsequently examined across all stages of the reproductive cycle, including pair formation, the nesting period, and fry care. Only one observer analyzed the videos. Given the lack of prior information about this species, we believe there was no bias influencing the behavioral recordings.

### Differential costs of oxidative stress between males and females

#### Collection of tissue samples

Tissue samples (5×5 mm) were excised from the dorsal and caudal fins of individuals that had been video-recorded. The total length of each fish was measured, after which individuals were returned to their original capture sites. Tissue samples were placed in Eppendorf tubes and transported to the laboratory in liquid nitrogen. It is important to note that the species we studied, *H. bartoni*, is endemic to Mexico and considered endangered. The population we studied represents the only known population worldwide, and it was not possible to collect blood samples without sacrificing the fish. Due to these conservation concerns, sacrificing fish or removing tissue from their body was not an option. Our best and ethically responsible choice was to sample a small fragment of the caudal fin. This approach allowed us to obtain sufficient biological material for analysis while minimizing harm and preserving the health and survival of these rare animals.

#### Obtaining enzymatic extracts

In the laboratory, fin samples from each fish were homogenized in 200 µl of PBS (Sigma-Aldrich) on ice using a motor-driven hand mixer (Cole-Parmer). The homogenates were subsequently centrifuged at 9402 ***g*** for 10 min at 4°C (Labnet International, Inc., Prism R), and the resulting supernatant was collected and stored at −70°C (Panasonic).

#### Determination of enzyme activities

Oxidative stress parameters were quantified using commercial assay kits in accordance with the manufacturers' instructions. The following antioxidant components were evaluated: total antioxidant capacity (TAC; BioVision), CAT (Invitrogen), and SOD (Sigma-Aldrich). In addition, prooxidant markers were assessed, including H_2_O_2_ (Invitrogen) and NO, the latter quantified indirectly via nitrite/nitrate levels (Sigma-Aldrich). Sample volumes were standardized according to the requirements of each assay kit. To prevent bias, all tests were conducted masked. One observer labeled the samples, while a different observer performed the oxidative stress analyses. Once all results were obtained, the sample identities were incorporated into the statistical analysis. We acknowledge that standardization using tissue instead of proteins may be less precise than protein-based standardization. However, this technique is permitted by the kit manufacturer.

### Data analyses

#### Behavior observations

For each reproductive stage (pair formation, nesting period, and fry care), the time spent by females and males on each behavior was quantified in seconds from video observations. To enable comparisons among videos of unequal duration, a proportional response variable (proportion of time) was calculated for each behavior as the ratio of time spent on a given behavior (s) to the total video duration (s). This variable, bounded between 0 and 1, was used as the primary response variable in subsequent analyses.

The proportional data exhibited a high frequency of zero values (i.e. behaviors not expressed during an observation). Accordingly, behaviors with fewer than 10% positive observations within each stage were excluded from statistical analyses and instead reported descriptively. This threshold was applied to reduce the risk of unstable parameter estimates arising from sparse data, excessive zero inflation, and potential separation in binomial models – issues commonly encountered when the number of observed events is low relative to the number of parameters estimated (Vittinghoff and McCulloch, 2007; Mansournia et al., 2018).

Analyses were conducted separately for each reproductive stage, as behavioral repertoires differ among pair formation, nesting, and fry care. Within each stage, all sufficiently frequent behaviors were analyzed jointly. Behavioral differences between males and females were evaluated using hurdle mixed-effects models, which partition two biological processes: (1) the probability of occurrence of a behavior and (2) the proportion of time allocated to that behavior when expressed. The occurrence component (presence/absence) was analyzed using a binomial generalized linear mixed model, whereas positive proportions were analyzed using beta mixed-effects models with a logit link function. Sex, behavioral category, and their interaction were included as fixed effects, and pair identity was incorporated as a random intercept to account for the nonindependence of individuals within pairs. As a complementary approach, zero-inflated beta mixed models were also fitted to assess the robustness of results when modeling zeros and positive proportions simultaneously.

Model selection was based on Akaike's information criterion (AIC), Bayesian information criterion (BIC), analysis of deviance (*D*^2^), and visual inspection of residuals. Pairwise post hoc comparisons were conducted using estimated marginal means with Tukey adjustment. Statistical significance was set at *P*<0.05. All analyses were performed in R (R Core Team, 2025; https://www.r-project.org/) using the glmmTMB (Brooks et al., 2017), DHARMa (Hartig, 2022; https://cran.r-project.org/package=DHARMa), emmeans (Lenth, 2023; https://CRAN.R-project.org/package=emmeans), performance (Lüdecke et al., 2021), tidyverse (Wickham et al., 2019), and ggplot2 (Wickham, 2016) packages. Dataset is provided as Dataset 1.

#### Differential costs of oxidative stress between males and females

Generalized linear models (GLMs) were used to evaluate differences in body size and oxidative stress variables between males and females. TAC, CAT, SOD, H_2_O_2_, and NO were analyzed separately for each reproductive stage. All models were fitted using a gamma distribution. Model selection was based on AIC, *D*^2^, and visual inspection of residuals. Pairwise post hoc comparisons among stages were conducted using Holm-adjusted tests. Statistical significance was set at *P*<0.05. Results are presented as mean±standard error. Analyses were conducted in R using the lme4 (Bates et al., 2015), DHARMa, glmmTMB (Brooks et al., 2017), gamlss (Rigby and Stasinopoulos, 2005), and ggplot2 (Wickham, 2016) packages.

#### Correlations between size and oxidative stress in females and males

To account for sex-specific differences in body size and oxidative stress variables, relationships among male and female size, TAC, and CAT activity were assessed using Spearman's rank correlations. Statistical significance was set at *P*<0.05. All analyses were performed in R using the ggpubr (Kassambara, 2023; https://cran.r-project.org/package=ggpubr) and corrplot (Wei and Simko, 2021; https://cran.r-project.org/web/packages/corrplot/index.html) packages.

## Supplementary Material



10.1242/biolopen.062571_sup1 Supplementary information

Dataset 1.
