# Supplementary material for: Oxidative stress: a sex-specific cost of parental care
Source: Biol Open. 2026 Jun 24;15(6):bio062571. doi: 10.1242/bio.062571 (PMC13382992; doi:10.1242/bio.062571)
Supplement: Supplementary information [file biolopen-15-062571-s1.pdf]

Table S1. Average frequency and proportion of time that male and female *H. bartoni* exhibited specific behaviors across three stages.

Behavior	Females		Males	
	Frequency of activity performance	Percentage of time spent on the activity (in seconds)	Frequency of activity performance	Percentage of time spent on the activity (in seconds)
**Pair formation**				
Chase	17	0.57 (62)	21	0.53 (58)
Confrontation	1	0.03 (3)	6	0.20 (22)
Flight	21	0.41 (45)	10	0.12 (13)
Synchronized swimming	63	1.45 (158)	63	1.36 (149)
Circular swim	66	0.89 (97)	66	0.89 (97)
Swim in turn	34	0.61 (67)	33	0.61 (67)
Approach	50	3.51 (383)	50	3.24 (354)
Cleaning	31	2.36 (258)	24	1.97 (215)
Feeding	64	6.29 (687)	37	4.08 (445)
Carved	0	0	9	0.24 (26)
They swam around the site	-	83.88 (9158)	-	86.76 (9472)
**Nesting period**				
Spawn	22	20.78 (594)	6	1.71 (49)
Chase	158	18.26 (522)	177	26.48 (757)
Inspection	46	14.76 (422)	71	31.02 (887)
Aeration	234	93.77 (2681)	96	16.51 (472)
Cleaning	239	64.25 (1837)	166	24.27 (694)

Biology Open • Supplementary information

Changing the location of the eggs	9	8.43 (241)	0	0
**Fry care**				
Chase	86	2.16 (197)	54	1.45 (132)
Confrontation	1	0.02 (2)	2	0.05 (5)
Inspection	22	3.34 (304)	36	3.13 (285)
Leave	0	0	6	2.28 (208)

Table S2. Results of the hurdle mixed-effects models evaluating behavioral differences during the pair formation stage in *H. bartoni*. Additionally, zero-inflated beta mixed models were fitted as a supplementary analysis to account for excess zeros.

The occurrence of behaviors was analyzed using a binomial model				
**Response variable**	**Explanatory variables**	**Estimate (Standard error)**	**z value**	**Pr(>|z|)**
	Intercept	0.663 (0.424)	1.562	0.118
**Circular swim**		1.153 (0.523)	2.206	**0.027**
**Swim in turn**		0.340 (0.477)	0.713	0.475
**Synchronized swimming**		0.586 (0.488)	1.202	0.229
**Cleaning**		-1.679 (0.479)	-3.499	**<0.001**
**Feeding**		0.340 (0.477)	0.713	0.475
**Chase**		-1.136 (0.464)	-2.446	**0.014**
**Flight**		-2.043 (0.497)	-4.112	**<0.001**
**Carved**		-3.004 (0.578)	-5.194	**<0.001**
**They swam around the site**		-17.972 (1082.369)	-0.017	0.986
**Sex**		0.-0.1691 (0.237)	-0.712	0.476
**Behavioral intensity (proportion of time) was analyzed using a beta mixed-effects model**				
**Conditional model**				
**Response variable**	**Explanatory variables**	**Estimate (Standard error)**	**z value**	**Pr(>|z|)**
	Intercept	-2.657 (0.212)	-12,497	**<0.001**
**Flight**		-1.868 (0.329)	-5.665	**<0.001**
**Synchronized swimming**		-1.485 (0.236)	-6.282	**<0.001**
**Circular swim**		-1.889 (0.231)	-8.157	**<0.001**
**Swim in turn**		-1.926 (0.240)	-8.011	**<0.001**
**Chase**		-1.635 (0.286)	-5.706	**<0.001**
**Cleaning**		-0.062 (0.328)	.0.192	0.848
**Feeding**		0.233 (0.290)	0.804	0.421
**Carved**		-1.789 (0.437)	-4.094	**<0.001**

Biology Open • Supplementary information

**Sex**		-0.059 (0.098)	-0.608	0.543
**Dispersion model**				
**Response variable**	**Explanatory variables**	**Estimate (Standard error)**	**z value**	**Pr(>|z|)**
	Intercept	2.550 (0.288)	8.835	**<0.001**
**Flight**		2.325 (0.536)	4.333	**<0.001**
**Synchronized swimming**		2.300 (0.389)	5.907	**<0.001**
**Circular swim**		2.7247 (0.393)	6.924	**<0.001**
**Swim in turn**		2.658 (0.409)	6.488	**<0.001**
**Chase**		2.039 (0.458)	4.445	**<0.001**
**Cleaning**		0.266 (0.509)	0.523	0.601
**Feeding**		-0.433 (0.400)	-1.084	0.278
**Carved**		2.109 (0.705)	2.991	0.002
**Zero-inflated beta mixed models**				
**Conditional model**				
**Response variable**	**Explanatory variables**	**Estimate (Standard error)**	**z value**	**Pr(>|z|)**
	Intercept	-2.705 (0.209)	-12.942	**<0.001**
**Flight**		-1.911 (0.332)	-5.754	**<0.001**
**Synchronized swimming**		-1.492 (0.242)	-6.158	**<0.001**
**Circular swim**		-1.849 (0.239)	-7.726	**<0.001**
**Swim in turn**		-1.854 (0.249)	-7.438	**<0.001**
**Chase**		-1.620 (0.294)	-5.500	**<0.001**
**Cleaning**		0.030 (0.313)	0.098	0.922
**Feeding**		0.266 (0.296)	0.901	0.367
**Carved**		-1.768 (0.411)	-4.295	**<0.001**
**Sex**		-0.017 (0.093)	-0.184	0.854
**Zero-inflation model**				
**Response variable**	**Explanatory variables**	**Estimate (Standard error)**	**z value**	**Pr(>|z|)**
	Intercept	-0.489 (0.291)	-1.680	0.092
**Flight**		1.642 (0.441)	3.723	**<0.001**

Biology Open • Supplementary information

**Synchronized swimming**	-0.454 (0.429)	-1.060	0.289
**Circular swim**	--0.896 (0.458)	-1.957	0.050
**Swim in turn**	-0.264 (0.420)	-0.628	0.529
**Chase**	0.895 (0.410)	2.182	**0.029**
**Cleaning**	1.336 (0.424)	3.150	**<0.001**
**Feeding**	-0.264 (0.420)	-0.628	0.529
**Carved**	2.482 (0.523)	4.739	**<0.001**
**Sex**	-0.017 (0.093)	-0.184	0.854

Bold values denote significant effects at p ≤ 0.05.

Biology Open • Supplementary information

Table S3. Results of the hurdle mixed-effects models evaluating behavioral differences during the nesting period in *H. bartoni*. Additionally, zero-inflated beta mixed models were fitted as a supplementary analysis to account for excess zeros.

**The occurrence of behaviors was analyzed using a binomial model**				
**Response variable**	**Explanatory variables**	**Estimate (Standard error)**	**z value**	**Pr(>|z|)**
	Intercept	2.601 (1.028)	2.530	**0.011**
**Inspection**		-0.742 (0.882)	-0.842	0.399
**Aeration**		-0.404 (0.906)	-0.447	0.655
**Chase**		20.645 (14643.244)	0.001	0.998
**Sex**		0.988 (0.737)	1.339	0.180
**Behavioral intensity (proportion of time) was analyzed using a beta mixed-effects model**				
**Response variable**	**Explanatory variables**	**Estimate (Standard error)**	**z value**	**Pr(>|z|)**
	Intercept	-1.682 (0.206)	-8.136	**<0.001**
**Inspection**		-1.326 (0.356)	-3.717	**<0.001**
**Aeration**		0.514 (0.262)	1.958	0.050
**Chase**		-1.437 (0.293)	-4.905	**<0.001**
**Sex**		-0.766 (0.304)	-2.514	**0.011**
	Sexmale:Inspection	0.898 (0.455)	1.974	**0.048**
	Sexmale:Aeration	-0.688 (0.419)	-1.641	0.100
	Sexmale:Chase	1.159 (0.397)	2.914	**0.003**
**Zero-inflated beta mixed models**				
**Conditional model**				
**Response variable**	**Explanatory variables**	**Estimate (Standard error)**	**z value**	**Pr(>|z|)**
	Intercept	-1.682 (0.206)	-8.136	**<0.001**
**Inspection**		-1.326 (0.356)	-3.717	**<0.001**
**Aeration**		0.514 (0.262)	1.958	0.050
**Chase**		-1.437 (0.293)	-4.905	**<0.001**
**Sex**		-0.766 (0.304)	-2.514	**0.011**

Biology Open • Supplementary information

	Sexmale:Inspection	0.898 (0.455)	1.974	**0.048**
	Sexmale:Aeration	-0.688 (0.419)	-1.641	0.100
	Sexmale:Chase	1.159 (0.397)	2.914	**0.003**
**Zero-inflation model**				
**Response variable**	**Explanatory variables**	**Estimate (Standard error)**	**z value**	**Pr(>|z|)**
	Intercept	-2.197 (0.608)	-3.610	**<0.001**
**Inspection**		0.587 (0.781)	0.752	0.451
**Aeration**		0.325 (0.811)	0.401	0.688
**Chase**		-19.423 (9042.309)	-0.002	0.998

Bold values denote significant effects at p ≤ 0.05.

Table S4. Results of the hurdle mixed-effects models evaluating behavioral differences during the fry care stage in *H. bartoni*. Additionally, zero-inflated beta mixed models were fitted as a supplementary analysis to account for excess zeros.

The occurrence of behaviors was analyzed using a binomial model				
**Response variable**	**Explanatory variables**	**Estimate (Standard error)**	**z value**	**Pr(>|z|)**
	Intercept	0.222 (0.535)	0.416	0.677
**Chase**		0.902 (0.695)	1.298	0.194
**Leave**		-2.979 (0.812)	-3.665	**<0.001**
**Sex**		1.336 (0.628)	2.127	**0.033**
**Behavioral intensity (proportion of time) was analyzed using a beta mixed-effects model**				
**Response variable**	**Explanatory variables**	**Estimate (Standard error)**	**z value**	**Pr(>|z|)**
	Intercept	-3.025 (0.297)	-10.166	**<0.001**
**Chase**		-0.792 (0.316)	-2.507	**0.012**
**Leave**		0.487 (0.531)	0.916	0.359
**Sex**		-0.131 (0.234)	-0.562	0.574
**Zero-inflated beta mixed models**				
**Conditional model**				
**Response variable**	**Explanatory variables**	**Estimate (Standard error)**	**z value**	**Pr(>|z|)**
	Intercept	-3.025 (0.297)	-10.166	**<0.001**
**Chase**		-0.792 (0.316)	-2.507	**0.012**
**Leave**		0.487 (0.531)	0.916	0.359
**Sex**		-0.131 (0.234)	-0.562	0.574
**Zero-inflation model**				
**Response variable**	**Explanatory variables**	**Estimate (Standard error)**	**z value**	**Pr(>|z|)**
	Intercept	-0.747 (0.404)	-1.847	0.064
**Chase**		-0.778 (0.638)	-1.220	0.222
**Leave**		2.539 (0.674)	3.762	**<0.001**

Bold values denote significant effects at $p \leq 0.05$.

Table S5. Results of the hurdle mixed-effects models evaluating behavioral differences across the three stages in *H. bartoni*. Additionally, zero-inflated beta mixed models were fitted as a supplementary analysis to account for excess zeros.

**The occurrence of behaviors was analyzed using a binomial model**				
**Response variable**	**Explanatory variables**	**Estimate (Standard error)**	**z value**	**Pr(>|z|)**
**Chase**	Intercept	-1.820 (1.581)	-1.151	0.249
	Nesting period	26.330 (15958.829)	0.002	0.998
	Fry care	5.548 (3.307)	1.678	0.093
	Sex	0.887 (0.830)	1.069	0.285
**Behavioral intensity (proportion of time) was analyzed using a beta mixed-effects model**				
**Response variable**	**Explanatory variables**	**Estimate (Standard error)**	**z value**	**Pr(>|z|)**
**Chase**	Intercept	-4.513 (0.265)	-17.015	**<0.001**
	Nesting period	1.205 (0.338)	3.567	**<0.001**
	Fry care	0.630 (0.350)	1.799	0.071
	Sex	-0.298 (0.236)	-1.258	0.208
	Sexmale:Nesting period	0.765 (0.307)	2.488	**0.012**
	Sexmale:Fry care	-0.023 (0.347)	-0.066	0.947
**Zero-inflated beta mixed models**				
**Conditional model**				
**Response variable**	**Explanatory variables**	**Estimate (Standard error)**	**z value**	**Pr(>|z|)**
**Chase**	Intercept	-4.513 (0.265)	-17.015	**<0.001**
	Nesting period	1.205 (0.338)	3.567	**<0.001**
	Fry care	0.630 (0.350)	1.799	0.071
	Sex	-0.298 (0.236)	-1.258	0.208
	Sexmale:Nesting period	0.765 (0.307)	2.488	**0.012**
	Sexmale:Fry care	-0.023 (0.347)	-0.066	0.947
**Zero-inflation model**				

Biology Open • Supplementary information

**Response variable**	**Explanatory variables**	**Estimate (Standard error)**	**z value**	**Pr(>|z|)**
**Chase**	Intercept	0.405 (0.288)	1.405	0.160
	Nesting period	-21.518 (7013.993)	-0.003	0.997
	Fry care	-1.931 (0.571)	-3.379	**<0.001**

Bold values denote significant effects at p ≤ 0.05.

Table S6. Results of the zero-inflated beta mixed models evaluating differences in total time allocated to parental care behaviors between females and males during the nesting and fry care stages in *H. bartoni*.

Zero-inflated beta mixed models				
**Conditional model**				
**Response variable**	**Explanatory variables**	**Estimate (Standard error)**	**z value**	**Pr(>|z|)**
	Intercept	0.011 (0.514)	0.023	0.982
**Stage-Fry care**		-0.067 (0.753)	-0.090	0.929
**Sex**		-1.412 (0.226)	-6.245	**<0.001**
**Sex:Stage-Fry care**		1.228 (0.283)	4.334	**<0.001**
**Zero-inflation model**				
**Response variable**	**Explanatory variables**	**Estimate (Standard error)**	**z value**	**Pr(>|z|)**
	Intercept	-22.81 (16411.65)	-0.001	0.999
**Stage-Fry care**		20.25 (16411.65)	0.001	0.999

Bold values denote significant effects at p ≤ 0.05.

Biology Open • Supplementary information

Table S7. Results of the generalized linear models (GLMs) evaluating variables across the three reproductive stages in *H. bartoni*.

Response variable	Explanatory variables	Estimate (Standard error)	z value	Pr(>|z|)
**Size**	Intercept	0.132 (0.003)	37.34	**<0.001**
	Nesting period	0.005 (0.003)	1.59	0.112
	Fry care	0.001 (0.004)	0.34	0.734
	SexMale	-0.025 (0.003)	-8.15	**<0.001**
**Total antioxidants**	Intercept	0.003 (0.0005)	7.367	**<0.001**
	Nesting period	0.001 (0.0005)	2.218	**0.026**
	Fry care	0.001 (0.0007)	1.756	0.079
	SexMale	-0.001 (0.0005)	-2.124	**0.033**
**Catalase**	Intercept	0.004 (0.0002)	15.548	**<0.001**
	Nesting period	-0.001 (0.0002)	-3.994	**<0.001**
	Fry care	-0.001 (0.0003)	-4.649	**<0.001**
	SexMale	0.0006 (0.0001)	3.374	**<0.001**
**Superoxide dismutase**	Intercept	0.0004 (0.00004)	19.299	**<0.001**
	Nesting period	0.0003 (0.0002)	1.471	0.141
	Fry care	0.0004 (0.0003)	1.657	0.097
	SexMale	-0.0002 (0.0002)	-1.358	0.174
**Hydrogen peroxide**	Intercept	0.0005 (0.00002)	18.371	**<0.001**
	Nesting period	0.00003 (0.00002)	1.471	0.141
	Fry care	0.00005 (0.00003)	1.657	0.097
	SexMale	0.00002 (0.00002)	-1.358	0.174
**Nitric oxide**	Intercept	0.620 (0.005)	116.98	**<0.001**
	Nesting period	-0.001 (0.006)	-0.20	0.842
	Fry care	-0.007 (0.006)	-1.06	0.289
	SexMale	-0.001 (0.005)	-0.30	0.761

Bold values denote significant effects at $p \leq 0.05$.

Dataset 1.

Available for download at

https://journals.biologists.com/bio/article-lookup/doi/10.1242/bio.062571#supplementary-data

Biology Open • Supplementary information
