# Supplementary material for: Oxidative stress: a sex-specific cost of parental care
Source: Biol Open. 2026 Jun 24;15(6):bio062571. doi: 10.1242/bio.062571 (PMC13382992; doi:10.1242/bio.062571)
Supplement: Supplementary file 1 [file biolopen-15-062571_review_history.pdf]

Oxidative stress: A sex specific cost of parental care

Norma Martínez-Lendech, Ivette Marai Villa-Villaseñor, Luis Felipe Mendoza-Cuenca and Jorge Contreras-Garduno
10.1242/bio.062571

Editor: Valentina di Santo

Review timeline
Original submission: 20 Marh 2026
Editorial decision: 2 April 2026
First revision received: 5 May 2026
Accepted: 14 May 2026

Original submission

First decision letter

MS ID#: bio.062571

MS Title: Oxidative stress: A sex specific cost of parental care

Authors: Norma Martínez-Lendech; Ivette Marai Villa-Villaseñor; Luis Felipe Mendoza-Cuenca; Jorge Contreras-Garduno

I have now reached a decision on the above manuscript.

The reviewer reports are shown at the bottom of this email.

As you will see, the reviewers raised a number of substantial criticisms that prevent me from accepting the paper at this stage.

The main issues to address are as follows:

Reviewer 1 raises concerns primarily about methodology. Behavioral data should be re-analyzed as proportions of observation time rather than raw durations, and pair identity should be incorporated into the statistical models to account for the non-independence of mates. The oxidative stress assays also require stronger justification for the use of fin tissue, clearer validation details, and normalization to protein concentration rather than sample volume.

Reviewer 2 raises concerns primarily about framing and interpretation. The definition of parental care should be clarified to distinguish pre-zygotic investment from post-zygotic care, the mechanistic link between parental behavior and oxidative stress needs fuller development, and alternative explanations for observed sex differences should be acknowledged. The Introduction should also be streamlined to remove repetitive material.

They suggest, however, that a revised version might prove acceptable, if you can address their concerns. If you think that you can deal satisfactorily with the criticisms on revision, I would be pleased to see a revised manuscript. We would then return it to the reviewers.

At this stage, we also ask you to ensure your manuscript complies with our formatting guidelines. Provided you are able to fully address the referees' comments, we are positive about publication of

© 2026. Published by The Company of Biologists under the terms of the Creative Commons Attribution License (https://creativecommons.org/licenses/by/4.0/).

your paper (we accept over 95% of revision submissions) and therefore hope you won't mind any extra work involved in reformatting your manuscript at this point.

Please upload both a 'clean' version of your Word file, along with a highlighted version clearly showing where you have made changes in the revised manuscript. Please avoid using 'Track changes' in Word files as these are lost in PDF conversion.

I should be grateful if you would also provide a point-by-point response detailing how you have dealt with the points raised by the reviewers in the 'Response to Reviewers' box. Please attend to all of the reviewers' comments. If you do not agree with any of their criticisms or suggestions please explain clearly why this is so.

Reviewer 1

Comments for the author

General Comments

The manuscript by Martínez-Lendech aims to quantify sex-specific differences in behavior and oxidative stress incurred during the reproductive period, including during pairing, nesting, and fry care in an threatened Central American cichlid Herichthys bartoni. The authors combine behavioral observations made with remote videos with direct measurements of antioxidant capacity through enzymatic activity assays and measurements of oxidative stress markers. This is an interesting study that may provide new insights into the metabolic stressors associated with parental care, and I commend the authors for the substantial amount of work undertaken to produce these results. However, there are several methodological issues that should be addressed before publication.

The authors collected relatively short videos (7-15 min, depending on reproductive stage), and they should clarify what proportion of each reproductive stage this observation period represents. I understand these recordings were intended as behavioral "snapshots," but the authors should justify why such brief observation periods are sufficient to characterize overall behavioral patterns. In addition, because video length appears to have varied among samples, behavioral data should be analyzed as the proportion of observation time spent on each behavior rather than as raw time, since raw duration will necessarily depend on the total length of the recording. Additional detail on the field site would also improve ecological context, including the density of breeding pairs and the presence and abundance of predators.

The authors may also wish to reconsider the behavioral analyses. As described, behavioral durations appear to be pooled within sexes and then analyzed for differences across reproductive stages. However, this approach does not account for the non-independence of mates within a pair since the behavior of one individual may be strongly influenced by that of its partner. If possible, the analyses should incorporate pair identity as a grouping factor and evaluate within-pair behavioral variation across stages and time points. I am not sure whether the sample size is sufficient for this approach, but it could provide valuable nuance in understanding behavioral variation among mating pairs. The authors should also state clearly whether repeated measures were present, for example whether the same pairs were recorded at multiple time points, and if so, whether this was accounted for statistically. If repeated measures could not be incorporated into the analysis, that limitation should be acknowledged explicitly.

There are several potential issues with the oxidative stress analyses that should be addressed, and the Methods require substantially more detail. The author's use of a fin clip is understandable given the protected status of the species, but fin tissue is not a standard target for these assays and may not reflect oxidative processes in more metabolically active tissues (as the authors note in the Discussion at line 558). Fin tissue composition (connective tissue, fin rays, mucus, and blood) may also affect the performance and interpretation of the assay kits used. The authors should describe how the kits were validated for fin homogenates, including the preparation of standard curves and serial dilutions to ensure that components of the fin clip tissue matrix are not interfering with the measurement.

© 2026. Published by The Company of Biologists under the terms of the Creative Commons Attribution License (https://creativecommons.org/licenses/by/4.0/).

The authors also appear to have standardized the assays by sample volume, which is not a valid way to ensure that comparable amounts of biological material were used across samples. Ideally, assay inputs should be standardized to protein concentration or, at minimum, to the mass of the original fin clip, rather than approximate size (5x5 mm as the authors state). Without appropriate normalization, variation in enzyme activities, H_2O_2, and indicators of NO may simply reflect differences in tissue amount, extraction efficiency, or other technical sources of variation rather than the biological effects described by the authors.

The authors should therefore provide a stronger justification for the use of fin clips, including support from studies in other species where possible, explain how fin clip based measurements relate to oxidative status in more metabolically active tissues, and include clear methodological details on sample normalization and assay validation for this tissue type.

Specific Comments

Lines 58-60: It would be more accurate to state that parental care benefits parental reproductive success and genetic fitness specifically, as it can also impose negative physiological costs, as discussed in the following section.

Line 127-129. The prediction should also include a statement on what the predicted differences in antioxidant capacity are.

Line 153. Behavior observations. Please include details on the number of pairs recorded for each stage and, if possible, whether pairs were recorded across multiple stages (i.e., repeated measures). Though, this may be impossible to know given the study design. Also, provide details on how much variation there was on the length of the videos within each time point.

Line 177. 2.4.1 Collection of tissue samples. Please include details on the number of fish captured during each reproductive period, and whether individuals were sampled multiple times. This may be possible to know given the existence of previous fin clips (though some regeneration may have occurred).

Line 192. 2.4.3 Determination of enzyme activities. Was total protein content of the homogenates also assessed? Enzyme activities and H_2O_2 and NO content should be standardized by protein content per sample. Standardizing volumes for each assay is not sufficient, as protein content may differ between samples due to technical differences in extraction efficiency that arise during the purification process.

Line 203, Section 2.5.1 Behavior observations: The authors state that "the proportions of time and the number of seconds that females and males spent on each behavior were compared," but the data appear to be presented, and presumably analyzed, as raw seconds. If video durations differed among recordings, behavioral data should be standardized as the proportion of total observation time spent on each behavior to avoid bias associated with unequal recording length. Then analyzed using methods appropriate for proportional data. In addition, the authors should clarify whether pair identity was considered in the analysis. Time spent on certain behaviors is likely influenced by the fish's mate and therefore may not be independent within a pair. If possible, the analyses should incorporate pair identity as a grouping factor and evaluate within-pair behavioral variation.

Line 236. 2.5.3 Correlations between size and oxidative stress in females and males. Is there a known relationship between fish size and antioxidant levels? Or was analysis done to test for one, rather than account for potential bias?

Figures 3-5: The figure captions state that only non-zero observations are shown. Please clarify why zero values were excluded from the presentation, as spending no time on a behavior is itself a biologically meaningful result. Presumably, zero observations were included in the statistical analyses give the test choice. In addition, please clarify whether the pairwise tests among behaviors were conducted on data averaged across sexes.

© 2026. Published by The Company of Biologists under the terms of the Creative Commons Attribution License (https://creativecommons.org/licenses/by/4.0/).

Lines 276-278: Why did the authors choose not to test for sex differences in time spent "guarding" when those data appear to be available? I understand that guarding is not listed as a behavior in the Table 2 ethogram, but it seems to be a metric the authors nevertheless recorded.

Lines 300-302: "However, sex significantly affected the probability of behavioral occurrence, with males exhibiting behaviors more frequently than females (Supplementary Table 4)." This result appears to be driven by the higher frequency of males "leaving." If so, it is unclear why this specific behavioral difference was not itself significant. Please clarify how this result was tested and how the overall sex effect should be interpreted in light of the apparent contribution of this single behavior.

Figure 7: The data presented here are confusing. I assume this figure shows the total time each individual spent performing all recorded behaviors within each reproductive stage, which appears to be relatively low during fry care. However, lines 294-295 state that "during the fry care stage, pairs spent 40% of their time in aquatic vegetation and 60% in unvegetated areas, consistently guarding their offspring." This seems inconsistent with the conclusion that little time was spent on parental behavior during fry care. As written, it appears somewhat arbitrary that only the behaviors listed in Table 2 are being counted as "behavior," while guarding or spatial association with offspring may be treated separately. The authors should clarify exactly what is included in this figure and reconsider how parental behavior is categorized and interpreted in this section.

Lines 347 - 350. Please include the units for total antioxidant levels here and for all enzyme measurements.

Line 370 -372. What is the rationale for not including data on superoxide dismutase, hydrogen peroxide levels, and nitric oxide levels in the main manuscripts? Readers will not want to look in the supplement. If this is a figure limit issue, then consider combining figures into multiple panels.

Lines 547-550: This statement appears to be inconsistent with the reported results, which indicate that males were larger. Please verify that this interpretation is correct. In addition, if this species has external fertilization, then referring to internal offspring development would not be appropriate. As written, the sentence is confusing and should be revised for clarity and biological accuracy.

Reviewer 2

Comments for the author

This manuscript describes an interesting study invesitgating sex-specific behavioural and physiological costs of parental care in Herichthys bartoni, combining field observations across reproductive stages with biochemical measures of oxidative stress. The integration of behavioural and physiological approaches in a natural population of an endangered species is a strength, and the study has the potential to make a valuable contribution to the literature on life-history trade-offs and parental investment. However, the manuscript in its current contains several issues that should be addressed:

1. The definition of parental care requires clarification. In lines 50-52, parental care is described as encompassing behaviours or physiological traits such as "egg production and incubation," yet egg production is more appropriately classified as reproductive investment rather than parental care. This inconsistency leads to confusion in the framing of costs throughout the manuscript. Even in the first paragraph in the intro, the definition seems to switch between behavioural only and also including physiological investment. The authors should clearly distinguish between pre-zygotic investment and post-zygotic parental care behaviours, and ensure that the terminology is applied consistently. Without this distinction, the subsequent arguments regarding the costs of parental care and their physiological correlates are often somewhat confusing.

2. The manuscript asserts that increased parental care leads to increased oxidative stress, but the mechanistic basis for this relationship is not sufficiently developed. For example, in lines 76-78, the authors state that increased parental care elevates cellular metabolism and reactive oxygen species

© 2026. Published by The Company of Biologists under the terms of the Creative Commons Attribution License (https://creativecommons.org/licenses/by/4.0/).

production, but the causal pathway is not clearly explained. Is it due to increased activity? It would strengthen the manuscript to explicitly link behavioural effort, such as increased activity during nest defense or egg aeration (and provide evidence from the literature that these are indeed energetically demanding), to metabolic demand and oxidative processes. The authors should also further explain why these processes are expected to differ between sexes in a predictable way.

3. The manuscript assumes a direct relationship between parental investment (presumably via physical activity but this isn't clear) and oxidative stress without adequately considering alternative explanations. Increased investment is implicitly linked to increased oxidative stress, but it is equally plausible that differences in oxidative status reflect variation in metabolic efficiency or oxidative defences with age, condition, or intrinsic physiological differences or efficiency between sexes. The authors should acknowledge these possibilities and adopt a more cautious interpretation of their results, particularly when attributing causality in the discussion but also where appropriate in the intro.

4. The Introduction is overly repetitive. For example, while the general background on parental care and oxidative stress is appropriate, similar points are reiterated multiple times (e.g. lines 69-82 and again in lines 88-94). The authors should streamline the intro by removing redundant material.

5. For the hypotheses presented in lines 126-129, it is unclear whether the expected sex differences in parental roles are already established for this species or are being tested here for the first time. If they are being observed for the first time, where do the sex-based predictions come from? I'm assuming from related species but this could be made clearer.

6. In lines 158-161, the number and duration of video recordings are described, but it is unclear how many unique pairs were observed, whether individuals were recorded multiple times, and how such potential non-independence was handled statistically. This is a critical issue, as repeated observations of the same individuals could violate assumptions of independence. The authors should clarify the sampling structure and, if necessary, incorporate appropriate statistical approaches to account for repeated measures.

7. Lines 166-169: it is not specified whether this process was manual or assisted by software, nor whether observer bias was assessed. Additional detail is needed regarding how behaviours were coded, whether observers were trained or blinded, and whether inter-observer reliability was evaluated.

8. The use of fin tissue for oxidative stress assays needs stronger justification. I get that you probably wanted to non-lethally sample this species, but fin tissue may not be the most metabolically active tissue and may therefore provide a limited representation of systemic oxidative stress associated with parental effort. This is discussed briefly in the discussion, which is good, but evidence that fin tissue is relevant for this sort of analyses should be provided, including any evidence that fin tissue is sensitive enough to detect differences arising from behavioural variation.

Additional Minor Comments

There are several minor issues that should be addressed. The coordinates in the Methods section (lines 136-138) should be checked (are y and O maybe left over from a Spanish translation?). The manuscript would also benefit from careful proofreading to improve grammatical clarity, for example in the Abstract (lines 21-22). Terminology should be used consistently throughout, particularly with respect to oxidative stress and oxidative balance.

Reviewer's Responses to Questions

Experimental quality
Does each figure have the proper controls?
If 'No', please indicate reasons in Comments for Author box below.
Reviewer #1:

© 2026. Published by The Company of Biologists under the terms of the Creative Commons Attribution License (https://creativecommons.org/licenses/by/4.0/).

- Yes

Reviewer #2:
- Yes

Were the data analyzed using appropriate statistical tests?
If 'No', please indicate reasons in Comments for Author box below.
Reviewer #1:
- No

Reviewer #2:
- Yes

Reproducibility
Were experiments performed using adequate number of biological replicates?
If 'No', please indicate reasons in Comments for Author box below.
Reviewer #1:
- Yes

Reviewer #2:
- Yes

Does the methods section provide sufficient detail to permit reproducibility?
If 'No', please indicate reasons in Comments for Author box below.
Reviewer #1:
- No

Reviewer #2:
- No

Completeness
Are the manuscript's conclusions supported by the data?
If 'No', please indicate reasons in Comments for Author box below.
Reviewer #1:
- No

Reviewer #2:
- Yes

Scholarship
Do the authors cite and discuss the merits of data that would argue for and against their conclusion?
If 'No', please indicate reasons in Comments for Author box below.
Reviewer #1:
- Yes

Reviewer #2:
- Yes

Does the manuscript title & abstract accurately reflect the contents of the manuscript, without hyperbole?
If 'No', please indicate reasons in Comments for Author box below.
Reviewer #1:

© 2026. Published by The Company of Biologists under the terms of the Creative Commons Attribution License (https://creativecommons.org/licenses/by/4.0/).

- Yes

Reviewer #2:
- Yes

First revision

Author response to reviewers' comments

We would like to thank you and the reviewers for your constructive feedback on our manuscript number: **bio.062571** entitled: "**Oxidative stress: A sex specific cost of parental care". **We have carefully considered each of your comments and made the necessary revisions accordingly. The feedback provided has significantly improved our manuscript. Below, we highlight in black the comments and our response in blue. In our revised manuscript, we also marked the changes incorporated based on suggestions from reviewer 1 in yellow and the reviewer 2 in green.
We are open to carrying out more changes if needed.

Reviewer #1:

Reviewer 1: Review of "Oxidative stress: A sex specific cost of parental care" by Martínez-Lendech et al

General Comments

The manuscript by Martínez-Lendech aims to quantify sex-specific differences in behavior and oxidative stress incurred during the reproductive period, including during pairing, nesting, and fry care in an threatened Central American cichlid Herichthys bartoni. The authors combine behavioral observations made with remote videos with direct measurements of antioxidant capacity through enzymatic activity assays and measurements of oxidative stress markers. This is an interesting study that may provide new insights into the metabolic stressors associated with parental care, and I commend the authors for the substantial amount of work undertaken to produce these results. However, there are several methodological issues that should be addressed before publication.

The authors collected relatively short videos (7-15 min, depending on reproductive stage), and they should clarify what proportion of each reproductive stage this observation period represents. I understand these recordings were intended as behavioral "snapshots," but the authors should justify why such brief observation periods are sufficient to characterize overall behavioral patterns. In addition, because video length appears to have varied among samples, behavioral data should be analyzed as the proportion of observation time spent on each behavior rather than as raw time, since raw duration will necessarily depend on the total length of the recording. Additional detail on the field site would also improve ecological context, including the density of breeding pairs and the presence and abundance of predators.

The authors may also wish to reconsider the behavioral analyses. As described, behavioral durations appear to be pooled within sexes and then analyzed for differences across reproductive stages. However, this approach does not account for the non-independence of mates within a pair since the behavior of one individual may be strongly influenced by that of its partner. If possible, the analyses should incorporate pair identity as a grouping factor and evaluate within-pair behavioral variation across stages and time points. I am not sure whether the sample size is sufficient for this approach, but it could provide valuable nuance in understanding behavioral variation among mating pairs. The authors should also state clearly whether repeated measures were present, for example whether the same pairs were recorded at multiple time points, and if so, whether this was accounted for statistically. If repeated measures could not be incorporated into the analysis, that limitation should be acknowledged explicitly.

There are several potential issues with the oxidative stress analyses that should be addressed, and the Methods require substantially more detail. The author's use of a fin clip is understandable given

© 2026. Published by The Company of Biologists under the terms of the Creative Commons Attribution License (https://creativecommons.org/licenses/by/4.0/).

the protected status of the species, but fin tissue is not a standard target for these assays and may not reflect oxidative processes in more metabolically active tissues (as the authors note in the Discussion at line 558). Fin tissue composition (connective tissue, fin rays, mucus, and blood) may also affect the performance and interpretation of the assay kits used. The authors should describe how the kits were validated for fin homogenates, including the preparation of standard curves and serial dilutions to ensure that components of the fin clip tissue matrix are not interfering with the measurement.

The authors also appear to have standardized the assays by sample volume, which is not a valid way to ensure that comparable amounts of biological material were used across samples. Ideally, assay inputs should be standardized to protein concentration or, at minimum, to the mass of the original fin clip, rather than approximate size (5x5 mm as the authors state). Without appropriate normalization, variation in enzyme activities, H2O2, and indicators of NO may simply reflect differences in tissue amount, extraction efficiency, or other technical sources of variation rather than the biological effects described by the authors.

The authors should therefore provide a stronger justification for the use of fin clips, including support from studies in other species where possible, explain how fin clip based measurements relate to oxidative status in more metabolically active tissues, and include clear methodological details on sample normalization and assay validation for this tissue type.

Response: We sincerely appreciate your thoughtful comments and the opportunity to clarify our methodology.

In this revised version, we clarify, point by point, the reasons for employing an interrupted focal method. Essentially, this was necessary because the fish spent the majority of their time concealed within the vegetation and only emerged for brief periods when they could be observed. Considering this comment, we incorporated the guarding behavior.

We agree that standardizing assays solely with fin may not be ideal. In our study, we carefully controlled both the body size of the animals and the size of the fin clips used (5x5 mm) to minimize technical variation that could influence the results. In addition, the oxidative stress kits employed in this study are designed for use with tissue or plasma samples. Plasma sampling was not feasible as it would require sacrificing the fish.

The use of tissue weight or controlled tissue size has been used in ecological questions. For example, Charradi et al. (2013; The journal of physiological sciences, 63, 530-541) emphasize the importance of controlling tissue mass to ensure that differences in oxidative stress biomarkers reflect biological variation rather than sample size disparities (Materials and Methods; Discussion sections). Similarly, Auberval et al. (2014; Diabetology & metabolic syndrome, 6, 157) highlight that normalizing oxidative stress markers to tissue mass is critical for accurate comparisons between experimental groups (Methods; Discussion).

Furthermore, in studies specific to fish biology and oxidative stress, Patnaik et al. (2023) standardized measurements by tissue weight when analyzing oxidative damage, underscoring that controlling tissue input is necessary to differentiate biological effects from technical variability (J Exp Biol 26:2558-2565). Likewise, Oliveira et al. (2014) explored oxidative costs related to social dominance in fish and controlled for tissue size/weight to accurately interpret oxidative stress levels between animals (J Exp Biol 217:2629-2638).

Additionally, it is important to note that the species we studied, *Herichthys bartoni,* is endemic to Mexico and considered endangered. The population we work with is the only known population in the world. Due to these conservation concerns, sacrificing fish or removing tissue from the body was not an option. Our best and ethically responsible choice was to sample a small fragment of the caudal fin. This approach allowed us to obtain sufficient biological material for analysis while minimizing harm and preserving the health and survival of these rare animals (lines 157-161). Although we could not measure protein concentration due to limited sample availability, we believe that controlling animal size and fin clip size provides a framework to ensure comparable biological material across samples, mitigating concerns about technical variation skewing enzyme activity, H_2O_2, or nitric oxide indicators. We also recognize limitations of our study (530-549).

© 2026. Published by The Company of Biologists under the terms of the Creative Commons Attribution License (https://creativecommons.org/licenses/by/4.0/).

Specific Comments

Lines 58-60: It would be more accurate to state that parental care benefits parental reproductive success and genetic fitness specifically, as it can also impose negative physiological costs, as discussed in the following section.

Response: We inserted this suggestion in lines 43-45.

Line 127-129. The prediction should also include a statement on what the predicted differences in antioxidant capacity are.

Response: We improved our hypothesis and predictions in lines 91-97.

Line 153. Behavior observations. Please include details on the number of pairs recorded for each stage and, if possible, whether pairs were recorded across multiple stages (i.e., repeated measures). Though, this may be impossible to know given the study design. Also, provide details on how much variation there was on the length of the videos within each time point.

Response: As suggested, we have included details on the number of pairs recorded at each stage, along with additional information regarding the rationale for video duration (lines 124-135). Specifically, 25 pairs were recorded during the pair formation stage (22–869 s), 15 pairs during the nesting period (491–1686 s), and 14 pairs during fry care (79–975 s).

Line 177. 2.4.1 Collection of tissue samples. Please include details on the number of fish captured during each reproductive period, and whether individuals were sampled multiple times. This may be possible to know given the existence of previous fin clips (though some regeneration may have occurred).

Response: Sample sizes are reported in lines 135-139. We ensured that previously observed pairs were not recaptured. This is now clarified in lines 135-139."

Line 192. 2.4.3 Determination of enzyme activities. Was total protein content of the homogenates also assessed? Enzyme activities and H_2O_2 and NO content should be standardized by protein content per sample. Standardizing volumes for each assay is not sufficient, as protein content may differ between samples due to technical differences in extraction efficiency that arise during the purification process.

Response: We kindly ask that you consider our previous response (see also lines 157-168 and 530-549). If this is deemed insufficient, we are willing to acknowledge this point as a limitation of the study. Alternatively, additional sampling could be conducted in a subsequent year; however, this would require repeating the study in its entirety.

Line 203, Section 2.5.1 Behavior observations: The authors state that "the proportions of time and the number of seconds that females and males spent on each behavior were compared," but the data appear to be presented, and presumably analyzed, as raw seconds. If video durations differed among recordings, behavioral data should be standardized as the proportion of total observation time spent on each behavior to avoid bias associated with unequal recording length. Then analyzed using methods appropriate for proportional data. In addition, the authors should clarify whether pair identity was considered in the analysis. Time spent on certain behaviors is likely influenced by the fish's mate and therefore may not be independent within a pair. If possible, the analyses should incorporate pair identity as a grouping factor and evaluate within-pair behavioral variation.

Response: Thank you for this comment. We agree that variation in video length could bias comparisons if only raw durations (in seconds) were analysed. Accordingly, Section 2.5.1 (see lines 196-226) has been revised to clarify that behavioural data were standardized as the proportion of total observation time devoted to each behaviour, calculated as the time spent performing a given behaviour divided by the total duration of the corresponding video. This proportional measure was used as the primary response variable in all statistical analyses.

© 2026. Published by The Company of Biologists under the terms of the Creative Commons Attribution License (https://creativecommons.org/licenses/by/4.0/).

Because these proportional data included a high frequency of zero values (i.e. behaviours not expressed in some observations), we employed statistical approaches appropriate for bounded, zero-inflated data in the 0–1 interval. Specifically, the main analyses were conducted using hurdle mixed-effects models, which separately evaluated (i) the probability of occurrence of a behaviour and (ii) the proportion of time allocated when the behaviour occurred. As a complementary analysis, zero-inflated beta mixed-effects models were also fitted.

We also appreciate the observation regarding potential non-independence within pairs. To account for the possibility that female and male behaviours within the same pair may be correlated, pair identity was included as a random effect (random intercept) in all mixed models. This approach allowed us to control for within-pair dependence while assessing behavioural differences and accounting for shared pair-level variation.

These clarifications and methodological revisions have been incorporated into the revised manuscript (see lines 196-226).

We sincerely appreciate your valuable comments, which have helped enhance the clarity and robustness of our results while leaving our discussion intact.

Line 236. 2.5.3 Correlations between size and oxidative stress in females and males. Is there a known relationship between fish size and antioxidant levels? Or was analysis done to test for one, rather than account for potential bias?

Response: We conducted this analysis to assess whether antioxidant levels are correlated with body size. Given that females were significantly smaller than males ($z = -8.15$, $p < 0.001$; males: 9.09 ± 0.17, females: 7.37 ± 0.13), differences in oxidative stress might have been influenced by size variation. Our results showed only a significant correlation between body size and total antioxidant capacity (see lines 366-371). Therefore, we propose that the observed differences are attributable to sex itself rather than to variation in body size.

Figures 3-5: The figure captions state that only non-zero observations are shown. Please clarify why zero values were excluded from the presentation, as spending no time on a behavior is itself a biologically meaningful result. Presumably, zero observations were included in the statistical analyses give the test choice. In addition, please clarify whether the pairwise tests among behaviors were conducted on data averaged across sexes.

Response: We agree that zero values are biologically meaningful, as they reflect instances when a particular behavior was not expressed during an observation period. In the original versions of Figures 3-5, only positive observations were shown to emphasize behavioral duration when the behavior occurred. However, we acknowledge that this approach did not fully represent the biological significance of zero observations. To address this, Figures 3–5 have been revised to include two complementary panels for each reproductive stage. Panel A illustrates the probability of occurrence of each behavior, based on the binomial component of the hurdle mixed-effects models, explicitly incorporating zero observations. Panel B depicts behavioral intensity, expressed as the proportion of total observation time allocated to a behavior when it occurred (i.e., positive observations only), corresponding to the positive beta component of the hurdle models.

Pairwise comparisons among behaviors were performed using estimated marginal means (EMMeans) derived from the fitted models. For overall comparisons among behaviors, results were averaged across sexes, as no significant sex differences or sex × behavior interactions were detected at this reproductive stage.

These clarifications, along with the corresponding figure revisions, have been incorporated into the revised manuscript (i.e. lines 851-878).

Lines 276-278: Why did the authors choose not to test for sex differences in time spent "guarding" when those data appear to be available? I understand that guarding is not listed as a behavior in the Table 2 ethogram, but it seems to be a metric the authors nevertheless recorded.

© 2026. Published by The Company of Biologists under the terms of the Creative Commons Attribution License (https://creativecommons.org/licenses/by/4.0/).

Response: Thank you for your observation. The values reported in lines 289-298 correspond to the behavioral category "inspection" as listed in Table 2, rather than representing an additional independent variable. This behavior involves individuals swimming around the spawning site, likely monitoring for potential intruders.

We acknowledge that our previous use of the phrase "guarding the nest" was imprecise and may have implied either an unreported metric or a behavior outside the ethogram categories.

To avoid any confusion, the text has been revised to consistently refer to this activity as "inspection of the spawning site," in accordance with the terminology used in Table 2. This behavior was also included in the formal statistical analyses comparing sexes and behavioral categories.

These clarifications and corrections have been incorporated into the revised manuscript.

Lines 300-302: "However, sex significantly affected the probability of behavioral occurrence, with males exhibiting behaviors more frequently than females (Supplementary Table 4)." This result appears to be driven by the higher frequency of males "leaving." If so, it is unclear why this specific behavioral difference was not itself significant. Please clarify how this result was tested and how the overall sex effect should be interpreted in light of the apparent contribution of this single behavior.

Response: Thank you for your insightful comment. We agree that the observed sex effect during the fry care stage is primarily attributable to the leave behavior, which was notably more frequent in males.

In the revised analysis, no significant sex differences were detected in behavioral intensity during this stage. However, sex significantly affected the probability of behavioral occurrence, with males showing a higher probability of expressing behaviors than females ($z = -2.12$, $p = 0.03$; Figure 5, Supplementary Table 4).

We have clarified this point in the revised Results and emphasized that this behavior should be interpreted with caution, given its low frequency and the large number of zero observations. Please refer to lines 312-317 for further details.

Figure 7: The data presented here are confusing. I assume this figure shows the total time each individual spent performing all recorded behaviors within each reproductive stage, which appears to be relatively low during fry care. However, lines 294-295 state that "during the fry care stage, pairs spent 40% of their time in aquatic vegetation and 60% in unvegetated areas, consistently guarding their offspring." This seems inconsistent with the conclusion that little time was spent on parental behavior during fry care. As written, it appears somewhat arbitrary that only the behaviors listed in Table 2 are being counted as "behavior," while guarding or spatial association with offspring may be treated separately. The authors should clarify exactly what is included in this figure and reconsider how parental behavior is categorized and interpreted in this section.

Response: Thank you for this comment. We agree that the presentation of this figure may have been unclear. Figure 7 represents the total time allocated to behavioral categories during the nesting period. For the fry care stage, the behaviors included were chase, inspection, and confrontation. The behavior leave was not included, as it reflects individuals moving away from the fry rather than a direct parental care action. Additionally, the variable guard was excluded from this figure because parental care could not be directly observed when both parents and fry were within the vegetation. Consequently, the figure may give the impression of reduced parental investment during this stage.

We also acknowledge that video durations varied among observations, which may have influenced comparisons based on absolute time. To address this issue, behavioral data have been standardized in the revised manuscript as the proportion of total observation time (our previous response). This approach enables more appropriate comparisons across stages and removes potential bias associated with variation in video duration. These clarifications have been incorporated into the revised manuscript.

© 2026. Published by The Company of Biologists under the terms of the Creative Commons Attribution License (https://creativecommons.org/licenses/by/4.0/).

Lines 347 - 350. Please include the units for total antioxidant levels here and for all enzyme measurements.

Response: We included the units in the text (i.e. lines 345, 352, 361).

Line 370 -372. What is the rationale for not including data on superoxide dismutase, hydrogen peroxide levels, and nitric oxide levels in the main manuscripts? Readers will not want to look in the supplement. If this is a figure limit issue, then consider combining figures into multiple panels.

Response: We did not include these figures because they were not statistically significant, and to our knowledge, only statistically significant results are typically presented in figures. However, these results are reported in the Results section (lines 360-362). If you believe that all results should be reported in a figure, we would be happy to do so.

Lines 547-550: This statement appears to be inconsistent with the reported results, which indicate that males were larger. Please verify that this interpretation is correct. In addition, if this species has external fertilization, then referring to internal offspring development would not be appropriate. As written, the sentence is confusing and should be revised for clarity and biological accuracy.

Response: We thank the reviewer for highlighting the error; indeed, the males are larger. We corrected the error and rewrote the paragraph to clarify the idea and the biological significance of the observed pattern (lines 428-432).

Reviewer 2:

This manuscript describes an interesting study invesitgating sex-specific behavioural and physiological costs of parental care in Herichthys bartoni, combining field observations across reproductive stages with biochemical measures of oxidative stress. The integration of behavioural and physiological approaches in a natural population of an endangered species is a strength, and the study has the potential to make a valuable contribution to the literature on life-history trade-offs and parental investment. However, the manuscript in its current contains several issues that should be addressed:

1. The definition of parental care requires clarification. In lines 50-52, parental care is described as encompassing behaviours or physiological traits such as "egg production and incubation," yet egg production is more appropriately classified as reproductive investment rather than parental care. This inconsistency leads to confusion in the framing of costs throughout the manuscript. Even in the first paragraph in the intro, the definition seems to switch between behavioural only and also including physiological investment. The authors should clearly distinguish between pre-zygotic investment and post-zygotic parental care behaviours, and ensure that the terminology is applied consistently. Without this distinction, the subsequent arguments regarding the costs of parental care and their physiological correlates are often somewhat confusing.

Response: Following the suggestion, we clarify the definition of parental care (lines 43-57).

2. The manuscript asserts that increased parental care leads to increased oxidative stress, but the mechanistic basis for this relationship is not sufficiently developed. For example, in lines 76-78, the authors state that increased parental care elevates cellular metabolism and reactive oxygen species production, but the causal pathway is not clearly explained. Is it due to increased activity? It would strengthen the manuscript to explicitly link behavioural effort, such as increased activity during nest defense or egg aeration (and provide evidence from the literature that these are indeed energetically demanding), to metabolic demand and oxidative processes. The authors should also further explain why these processes are expected to differ between sexes in a predictable way.

Response: We hope that our revised introduction better supports our arguments (59-78). For example, you are correct that parental care increases an organism's activity and imposes metabolic demands (see lines 59–67). In lines 69–78, we further explain why we expect sexual differences.

© 2026. Published by The Company of Biologists under the terms of the Creative Commons Attribution License (https://creativecommons.org/licenses/by/4.0/).

3. The manuscript assumes a direct relationship between parental investment (presumably via physical activity but this isn't clear) and oxidative stress without adequately considering alternative explanations. Increased investment is implicitly linked to increased oxidative stress, but it is equally plausible that differences in oxidative status reflect variation in metabolic efficiency or oxidative defences with age, condition, or intrinsic physiological differences or efficiency between sexes. The authors should acknowledge these possibilities and adopt a more cautious interpretation of their results, particularly when attributing causality in the discussion but also where appropriate in the intro.

Response: Considering the reviewer's comment, we modified the introduction and discussion to incorporate alternative interpretations of the observed patterns, particularly to avoid implying causality when we do not have sufficient evidence for such assertions. See for example lines 530-549.

4. The Introduction is overly repetitive. For example, while the general background on parental care and oxidative stress is appropriate, similar points are reiterated multiple times (e.g. lines 69-82 and again in lines 88-94). The authors should streamline the intro by removing redundant material.

Response: We improved the introduction. Lines 41-97.

5. For the hypotheses presented in lines 126-129, it is unclear whether the expected sex differences in parental roles are already established for this species or are being tested here for the first time. If they are being observed for the first time, where do the sex-based predictions come from? I'm assuming from related species but this could be made clearer.

Response: As part of the revisions to the introduction, we have aimed to clarify the rationale underlying our hypothesis. We emphasize that both our preliminary observations and existing literature on biparental cichlids indicate that differential sexual roles during parental care are commonly observed (lines 69-78). Given the rarity of this species, our study represents the first comprehensive documentation of its courtship, parental care, and physiology.

6. In lines 158-161, the number and duration of video recordings are described, but it is unclear how many unique pairs were observed, whether individuals were recorded multiple times, and how such potential non-independence was handled statistically. This is a critical issue, as repeated observations of the same individuals could violate assumptions of independence. The authors should clarify the sampling structure and, if necessary, incorporate appropriate statistical approaches to account for repeated measures.

Response: Both reviewers raised a similar concern, and we have accordingly revised the relevant section to clarify the number and duration of the videos (lines 122–139). We also refer you to our responses X, Y, and Z to Reviewer 1. Furthermore, we have clarified this aspect in methods as follows: "After completing the behavioral observations for each pair, individuals were captured to obtain measurements and fin clips for oxidative stress analyses (see below). This procedure enabled us to confirm the identities of previously observed individuals, thereby preventing their reuse and avoiding pseudoreplication. In total, we sampled 54 unique pairs."

7. Lines 166-169: it is not specified whether this process was manual or assisted by software, nor whether observer bias was assessed. Additional detail is needed regarding how behaviours were coded, whether observers were trained or blinded, and whether inter-observer reliability was evaluated.

Response: To minimize potential bias arising from inter-observer variability, all behavioral assessments were conducted manually from video recordings by a single, well-trained observer. Each video was first reviewed to document female behavior, followed by a second review to record male behavior, ensuring a consistent and uniform evaluation across all observations. Lines 149-151 and 187-190.

8. The use of fin tissue for oxidative stress assays needs stronger justification. I get that you probably wanted to non-lethally sample this species, but fin tissue may not be the most

© 2026. Published by The Company of Biologists under the terms of the Creative Commons Attribution License (https://creativecommons.org/licenses/by/4.0/).

metabolically active tissue and may therefore provide a limited representation of systemic oxidative stress associated with parental effort. This is discussed briefly in the discussion, which is good, but evidence that fin tissue is relevant for this sort of analyses should be provided, including any evidence that fin tissue is sensitive enough to detect differences arising from behavioural variation.

Response: Thank you for this important comment. We agree that the choice of tissue requires clear justification. Fin tissue was selected primarily due to its non-lethal and minimally invasive nature, which is especially critical when working with endemic species where minimizing impact on individual organisms is paramount (we incorporated the lines 157-168).

Although fin tissue exhibits lower metabolic activity compared to organs such as liver or muscle, it is well-vascularized and physiologically responsive. It has been validated as a reliable source of biochemical and molecular biomarkers in fish. Previous studies have shown that peripheral tissues, including fin clips, can accurately reflect systemic oxidative status and respond to environmental and physiological stressors, such as those related to behavioral activity and energetic demands (e.g., Zhang et al., 2022; Toxics, 10).

Additional Minor Comments
There are several minor issues that should be addressed. The coordinates in the Methods section (lines 136-138) should be checked (are y and O maybe left over from a Spanish translation?). The manuscript would also benefit from careful proofreading to improve grammatical clarity, for example in the Abstract (lines 21-22). Terminology should be used consistently throughout, particularly with respect to oxidative stress and oxidative balance.

Response: We are sorry, we translated the lines 104-106. We improved the abstract (lines 19-38) and reviewed the MS. The terminology was corrected.

Second decision letter

MS ID#: bio.062571R1

MS Title: Oxidative stress: A sex specific cost of parental care

Authors: Norma Martínez-Lendech; Ivette Marai Villa-Villaseñor; Luis Felipe Mendoza-Cuenca; Jorge Contreras-Garduno

I am happy to tell you that your manuscript has been accepted for publication in Biology Open, pending our standard publication integrity checks. It was accepted on 14th May 2026.

© 2026. Published by The Company of Biologists under the terms of the Creative Commons Attribution License (https://creativecommons.org/licenses/by/4.0/).
